# Chromosome architecture constrains horizontal gene transfer in bacteria

**DOI:** 10.1371/journal.pgen.1007421

**Published:** 2018-05-29

**Authors:** Heather L. Hendrickson, Dominique Barbeau, Robin Ceschin, Jeffrey G. Lawrence

**Affiliations:** 1 Department of Biological Sciences, University of Pittsburgh, Pittsburgh, Pennsylvania, United States of America; 2 Institute of Natural and Mathematical Sciences, Massey University, Auckland, New Zealand; Imperial College London, UNITED KINGDOM

## Abstract

Despite significant frequencies of lateral gene transfer between species, higher taxonomic groups of bacteria show ecological and phenotypic cohesion. This suggests that barriers prevent panmictic dissemination of genes via lateral gene transfer. We have proposed that most bacterial genomes have a functional architecture imposed by Architecture IMparting Sequences (AIMS). AIMS are defined as 8 base pair sequences preferentially abundant on leading strands, whose abundance and strand-bias are positively correlated with proximity to the replication terminus. We determined that inversions whose endpoints lie within a single chromosome arm, which would reverse the polarity of AIMS in the inverted region, are both shorter and less frequent near the replication terminus. This distribution is consistent with the increased selection on AIMS function in this region, thus constraining DNA rearrangement. To test the hypothesis that AIMS also constrain DNA transfer between genomes, AIMS were identified in genomes while ignoring atypical, potentially laterally-transferred genes. The strand-bias of AIMS within recently acquired genes was negatively correlated with the distance of those genes from their genome’s replication terminus. This suggests that selection for AIMS function prevents the acquisition of genes whose AIMS are not found predominantly in the permissive orientation. This constraint has led to the loss of at least 18% of genes acquired by transfer in the terminus-proximal region. We used completely sequenced genomes to produce a predictive road map of paths of expected horizontal gene transfer between species based on AIMS compatibility between donor and recipient genomes. These results support a model whereby organisms retain introgressed genes only if the benefits conferred by their encoded functions outweigh the detriments incurred by the presence of foreign DNA lacking genome-wide architectural information.

## Author summary

The potential success of horizontal gene transfer events is historically equated to the benefits conferred by encoded products. Here we show that gene transfer events are observed less frequently if the introduced genes disrupt important patterns of genomic information, suggesting that this disruption would confer an unacceptable cost. As a result, gene transfer events are less likely to be successful if the potential donor genomes have incompatible genome architecture. Because more distantly-related genes are less compatible, chromosome architecture serves as a mechanism to bias gene transfer events to those involving closer relatives, thereby providing a mechanism for the genotypic and phenotypic cohesion of higher taxonomic groups.

## Introduction

The evolutionary histories of genes within bacterial genomes have long been shown to be highly incongruent [1]; [2–4]. Horizontal Gene Transfer (HGT) between species enables bacteria to acquire and potentially utilize any gene that it encounters in the biosphere, thus catalysing exploration of novel niches, the evolution of pathogenicity, or responses to environmental stressors in manners beyond the capabilities of their ancestors. While the amount of transferred DNA inferred in individual genomes varies depending on methodology for detection, the age limit for distinguishing between acquired and native genes, and the taxa involved, the fraction of bacterial genomes resulting from recent transfer is very large, ranging from 20% to 80% of the genome [5–7]. Yet despite the preponderance and pervasiveness of this genetic admixture [8,9], members of higher taxonomic groups share large degrees of genotypic and phenotypic similarity [4,10] which belie the potential for genome homogenization between groups afforded by such rampant transfer.

This cohesion within groups indicates that more closely related bacterial groups are more likely to exchange genes successfully [9], resulting in genotypic similarity due to shared pathways for gene trafficking, rather than a common pool of unchanging ancestral genes. Two mechanisms could result in the preferential use of gene donors: either bacteria are predominantly exposed to incoming DNA from closely-related taxa, or genes from related taxa are preferentially retained following their introduction [11,12]. For example, similarity in GC content [11] or ecological niche [4,13,14] between inferred donor and recipient genomes are proposed to influence HGT success. While organisms dwelling in the same environment likely have increased opportunities for gene exchange (owing to the increased rate of both direct or indirect encounters among organisms in closer proximity) and carry genes which are useful in that setting; these communities contain many unrelated taxa and do not necessarily bias gene transfer towards related members. Given the paucity of genes recalcitrant to HGT [15], these factors alone are insufficient to reconcile the disparity between the scope and frequency of gene transfer, its role in promoting niche invasion, and overall levels of similarity among higher taxonomic groups of bacteria.

Any benefits conferred by horizontally acquired genes that favor their retention must exceed any detriments imparted by the integration of incompatible foreign DNA into an evolutionarily coadapted genome. We have previously drawn attention to molecular mechanisms by which integrated DNA can negatively impact recombinant survival [8]. This constraint centers on the role of Architecture IMparting Sequences (AIMS), strand-biased repetitive elements which act during DNA segregation. The improper distribution of these sequences in newly-acquired genes should disrupt AIMS-based genome architecture, and thus negatively impact cellular fitness; such genes would be preferentially lost if the encoded functions were insufficiently beneficial to overcome this detriment. If AIMS were shared among more closely-related taxa, they could reinforce cohesion within bacterial clades by counter-selecting gene acquisition from distantly-related taxa which do not have the sequences in congruent distributions. This makes AIMS distinct from other conserved features in chromosomes such as gene orientation, rRNA location, Chi sites or Ter sequences which will impose selective constraints but do not have the qualities of abundance or variation between taxa that would arbitrate the success of transfer events.

**Fig 1:**
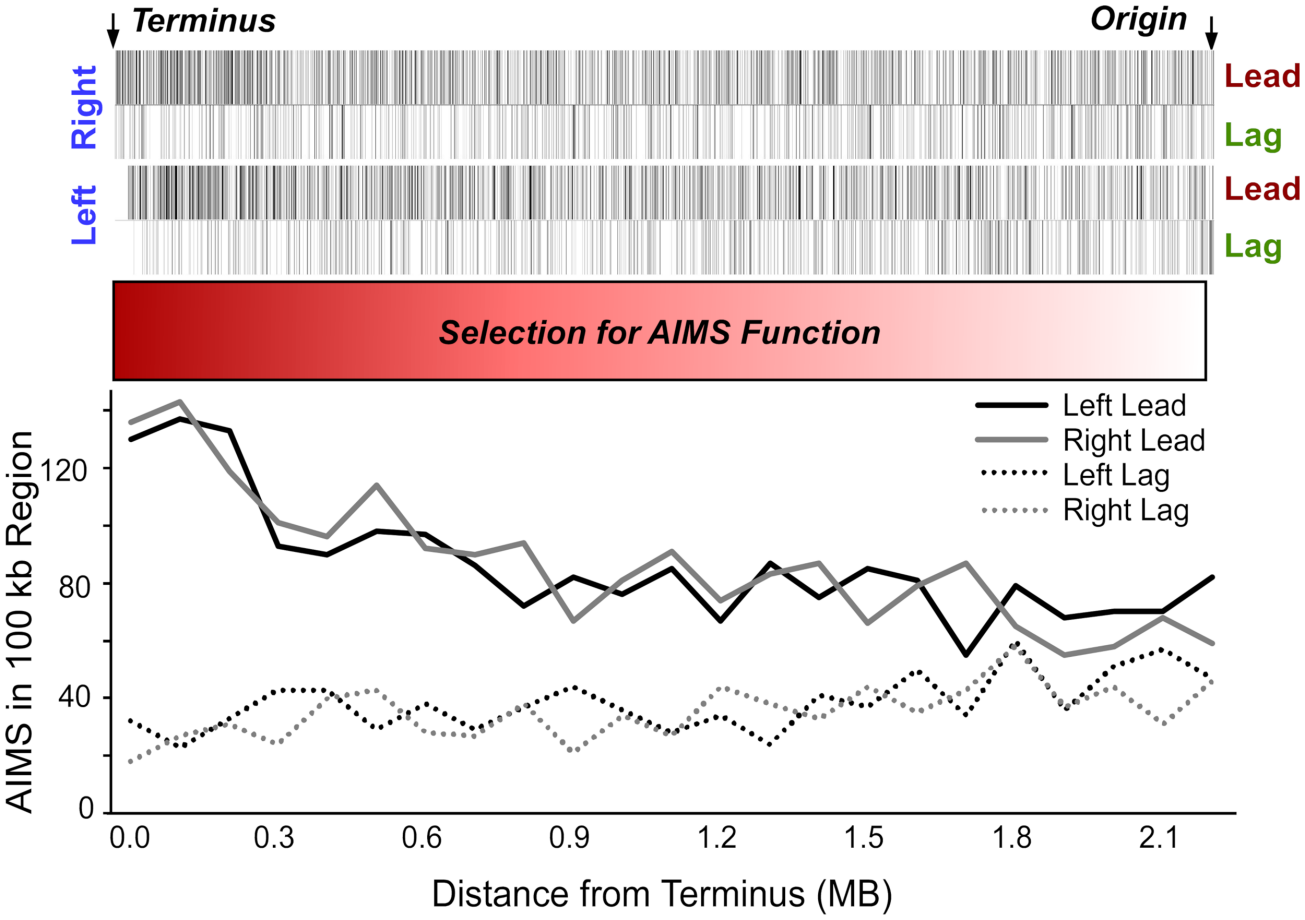
The distribution of 7119 copies of 27 AIMS in the *Escherichia coli* MG1655 chromosome. Octamers are represented as hash marks and plotted by position relative to the replication origin and terminus. AIMS increase in both abundance and strand-bias from the origin to terminus, reflecting a gradient of selection for their function; increased selection for AIMS function is denoted by darker red.

AIMS form the basis of an architecture present in nearly all bacterial genomes [16,17]. Chromosomes are immense polymers with embedded instructions that direct faithful replication, repair, defense and segregation [18]. AIMS are identified as strand-biased octamers which, unlike simple strand-biased sequences such as chi [19], increase in abundance and degree of strand-bias with proximity to the replication terminus (Fig 1)[16]. This pattern suggests that selection for AIMS function would be maximal at the replication terminus (Fig 1) [16]. AIMS are proposed to aid in processes such as DNA replication, repair and segregation [16]; for example, FtsK Orienting Polar Sequences (KOPS) are AIMS that assist the directional loading of the FtsK translocase, which pumps chromosomes trapped in division septa into the proper daughter cells [20–24]. The functions of most AIMS are unknown, and AIMS serve as surrogates for the true targets of selection. Detrimental effects of changing AIMS from permissive (on leading strand) to non-permissive (on lagging strand) orientations have been observed in *E*. *coli* [25]. Suites of AIMS are similar in sequence among more closely-related taxa [16,26], suggesting that clades of bacteria share AIMS architectures.

We propose that disruption of genome-wide AIMS organization will have deleterious effects. For example, inversions restricted to a single chromosome arm can place potentially large numbers of AIMS into nonpermissive orientations; therefore, we predict that the size and frequency of inversions will be correlated with distance from the replication terminus, as inversions close to the terminus would place AIMS in their nonpermissive orientations where selection for their function is the greatest. Similarly, insertion of foreign DNA will be detrimental if AIMS in the recipient organism are not strand-biased in the donor genome, thereby precluding introgressed fragments from bearing AIMS in predominantly permissive orientations. We predict that the degree to which newly-acquired DNA carries AIMS in their permissive orientation will also be negatively correlated with distance from the terminus. If so, then these results would validate the role of AIMS in promoting gene transfer among organisms wherein AIMS are shared, or at least strand-biased, among members of the same clade. Herein, we demonstrate that these predictions are validated and propose a framework for interspecific gene transfer based on AIMS compatibility.

## Results and discussion

### AIMS are under selection in bacterial genomes

AIMS are identified as degenerate octamers with three properties: (i) they are strand-biased, with more instances appearing on leading strands than on lagging strands, (ii) their abundance on leading strands increases on both chromosome arms with distance from the replication origin (proximity to the replication terminus or telomere), and (iii) their degree of strand-bias also increases with distance from the replication origin. The increase in strand-bias and abundance with proximity to the terminus reflects selection for this gradient as it cannot be explained by mutational processes [27]. Oligomers identified with these properties often fall into groups of related or overlapping octamers, likely reflecting selection on a longer, degenerate sequence. However, small numbers of sequences with these properties may arise by stochastic factors alone.

To identify sets of potential AIMS which minimize the number of sequences arising by stochastic processes, we first identified replication breakpoints in bacterial genomes using a Markov approach (see Methods) since AIMS are strand biased and required known replication breakpoints to identify; the breakpoints were classified as either a replication origin or terminus so that the majority of genes are transcribed from leading strands [28,29]. The location of the terminus was refined and validated using the locations of putative *dif* sites [30]; the predicted termini and the annotated *dif* sites were very close (S5 Table), providing confidence that both the replication origin and terminus were predicted accurately. Recently-recombined regions were identified by comparison with closely-related genomes and removed, leaving the ancestral sequences whose properties reflect consistent mutational biases. The numbers of AIMS-like oligomers were identified in this ancestral backbone using a range of criteria, including different degrees of overall strand-bias and different degrees of increase in abundance with proximity to the replication terminus (S1 Dataset). As expected, the numbers of potential AIMS decrease as the criteria for their selection become more stringent.

**Fig 2:**
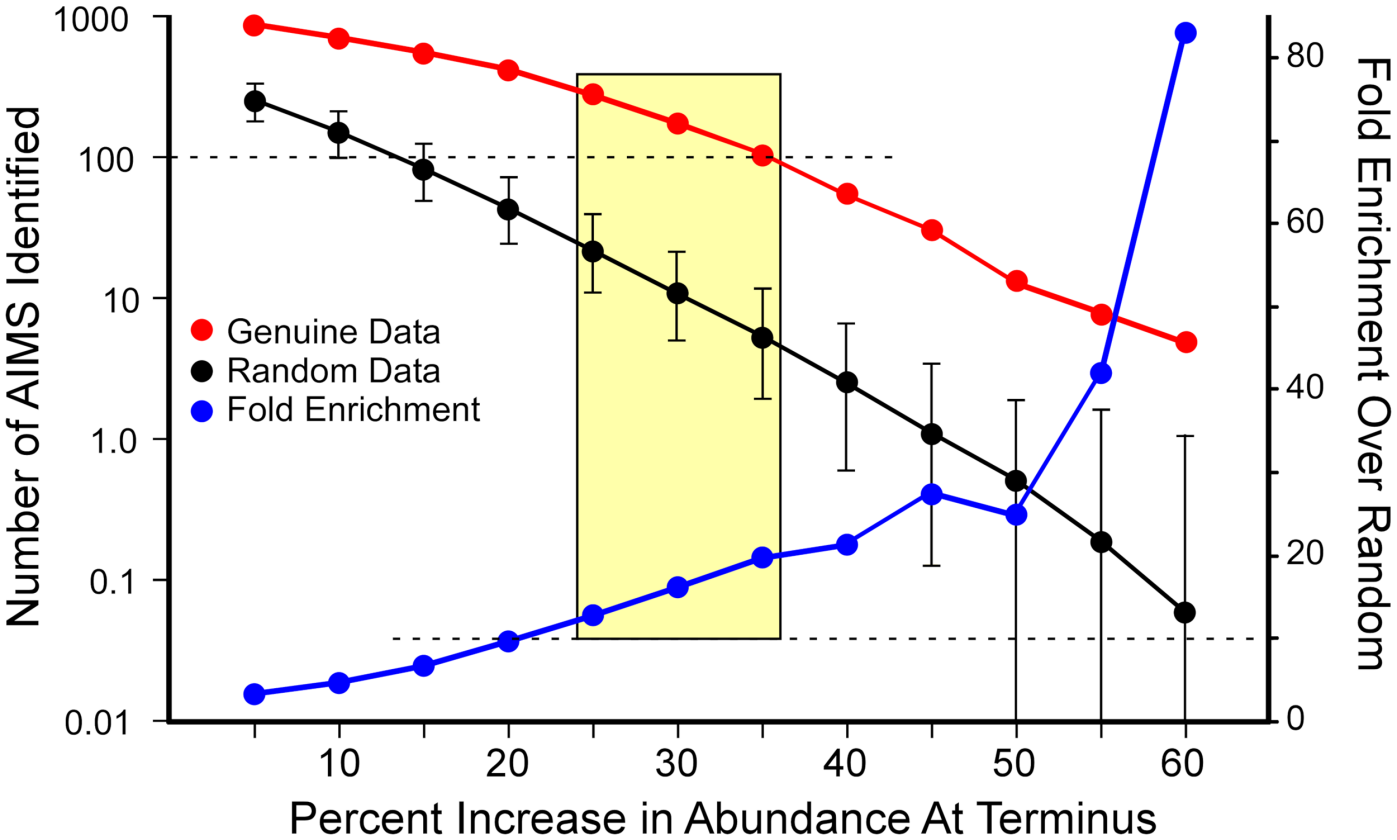
Establishing criteria for the identification of putative AIMS in the *Escherichia coli* genome. AIMS were identified as octamers (degenerate at up to 2 positions) with at least 70% strand-bias, present in at least 96 copies per genome, and with the indicated percent increase in abundance in the terminus-proximal region. As expected, fewer putative AIMS are identified as stringency increases. Genuine data are shown in red; the numbers of AIMS detected in genomes wherein fragments were randomized within chromosome arms are shown in black (mean +/- 2 standard deviations in 100 replicates). The blue curve shows the fold enrichment of AIMS in genuine genomes compared with randomized genomes. The shaded area depicts settings wherein AIMS are abundant (>100 different AIMS identified) and enriched at least 10-fold in genuine genomes relative to randomized controls.

To determine what fraction of oligomers reflects selection for function (true AIMS), the same process was implemented on the backbone genomes after the positions of 10 kb segments were randomized within chromosome arms. This randomization preserved overall strand-bias, but eliminated any result of a gradient of selection from origin to terminus; putative “AIMS” identified within such randomized genomes would be the result of stochastic factors alone. As expected, fewer putative AIMS are identified in randomized genomes as compared to genuine genomes (Fig 2). Suitable selection criteria are defined as those wherein the numbers of putative AIMS are at least 10-fold higher in the genuine genome as compared to those identified in randomized genomes so that at least 91% of the octamers identified in genuine genomes are true AIMS, reflecting selection rather than stochastic processes. In this way, we are confident that the sets of AIMS we identified reflect the action of selection, with minimal numbers of confounding sequences.

### Inversions are constrained within genomes as predicted by AIMS

If the distribution of AIMS is maintained by selection, then genome rearrangements which disrupt these distributions will be counter-selected. Inversions are reported to be non-random with respect to the origin and terminus [31]. Inversions that do not include either the replication origin or terminus will move AIMS that were formerly in their permissive orientations into their nonpermissive orientations, and thus should be counter-selected. Therefore, we predict that inversions observed in extant genomes will become both smaller and less frequent with proximity to the replication terminus, where selection for AIMS function is maximal (Fig 1).

We identified inversions in 159 pairs of genomes from 43 families representing 17 divisions of bacteria (S1 and S2 Tables); inversions that included the replication origin or terminus were ignored as they do not affect the strand-bias of AIMS. Genes were identified using the annotation provided; orthologous genes were identified as reciprocal best BLAST hits, where genes were aligned over >85% of their length. Inversions were identified as groups of orthologous genes that had been reversed in orientation relative to proximal, otherwise syntenic genes in a closely related genome (see Materials & methods). In total, 634 unique inversions were identified; inversion positions were defined as the percentage of genome distance from the replication terminus to the center of the inversion, averaged between the two genomes compared.

**Fig 3:**
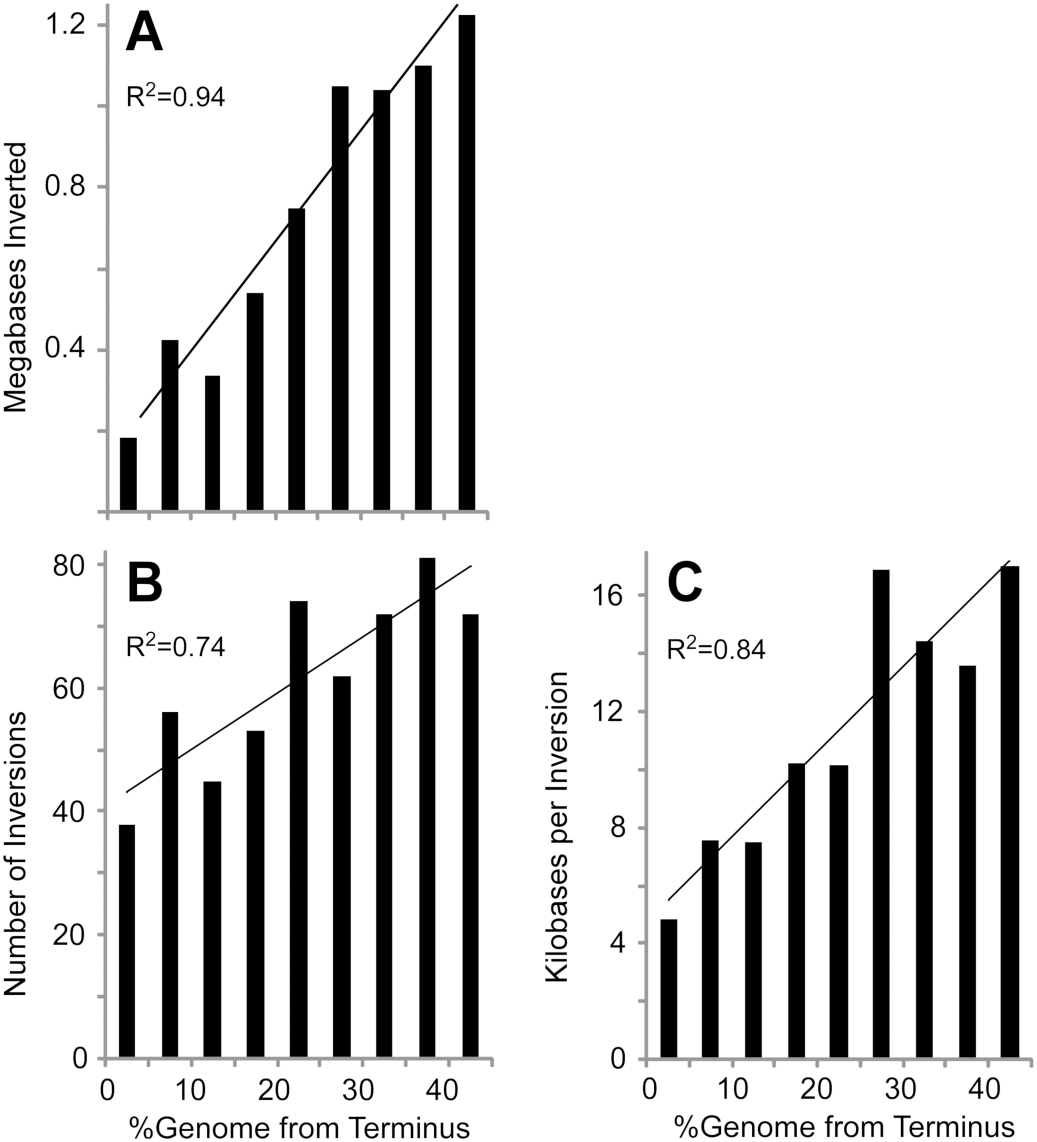
Distribution of inversions in completely sequenced bacterial genomes. A total of 634 inversions were identified in 159 pairwise comparisons of 214 separate completely sequenced genomes (See S2 Table for details). All data are plotted as % genome distance of the midpoint of the inversion from the replication terminus. **A**. The total length of DNA inverted plotted by genome position across all genomes included in the analysis. **B**. The number of individual inversions plotted by genome position across all genomes included in the analysis. **C**. The average size of the individual inversions plotted by genome position.

The distribution of inversions within bacterial chromosomes shows a clear and unambiguous relationship with respect to the replication terminus (Fig 3). As predicted by the distribution of AIMS, the number of inversions observed in genome alignments is strongly positively correlated with distance from the replication terminus (Fig 3B; R = 0.86). Moreover, the length of observed inversions is also strongly positively correlated with the distance from the replication terminus (Fig 3C; R = 0.92). Taken together, six times as much inverted DNA is found near the replication origin as compared to the replication terminus (Fig 3A; R = 0.97).

In addition to typifying the data set as a whole, this pattern is evident within subsets of genomes with different properties. For example, inverted DNA is clearly lacking from the region of the replication terminus in different taxonomic groups including Actinobacteria, α-proteobacteria, γ-proteobacteria, δ,ε-proteobacteria, and Firmicutes (S1 Fig), in genomes from low (35%) to high (75%) %GC (S1 Fig), and in genomes ranging from 2 MB to 9.5 MB in size (S1 Fig). Only small, AT-rich genomes failed to show a positive relationship between the amount of inverted DNA and distance from the terminus (S1 Fig); these organisms are primarily intracellular parasites whose genomes show weak purifying selection and high rates of chromosomal rearrangement [32,33], which would occlude any pattern we would hope to detect.

Rather than reflecting constraints imposed by AIMS, the decrease of inversion size and frequency with proximity to the replication terminus could reflect a preference for the individual genes to be transcribed from a particular strand [34,35]. For example, highly-expressed genes are more often transcribed from leading strands, thus avoiding collisions between DNA- and RNA-polymerases. If highly-expressed genes were found preferentially near the terminus, our results would be observed. To test this hypothesis, we used the degree of codon selection as a surrogate metric for average level of gene expression [36]. We calculated codon usage bias using four separate metrics within 12 representative genomes from 5 divisions of bacteria. In most genomes, codon usage bias was not correlated with distance from the replication terminus (S6 Table); in the few genomes which show weak effects, codon usage bias increased with proximity to the replication origin, not the replication terminus (S6 Table). This is unsurprising, as highly-expressed genes in many organisms are found close to the replication origin, likely because of the higher average ploidy numbers there [19,37,38]. Therefore, we reject the hypothesis that inversions are avoided near the terminus because the genes in that region are more highly expressed.

Alternatively, the dearth of inversions in the terminus region could reflect a gradient in the distribution of the small, repeated sequences that catalyze inversion formation [39–41]. To test this, we examined the spacing between adjacent inverted pentamers, hexamers and heptamers within each chromosome arm and regressed the average spacing for 10kb intervals against distance of the interval from the terminus (S6 Table). While these oligomer lengths are not equal to those observed for spontaneous inversion join points [41], their greater numbers allow for a more robust analysis while being able to capture any trend that would impact the slightly longer repeats observed. The distribution of the oligomers we examined showed no change in abundance near the replication terminus (S6 Table); therefore, we reject the hypothesis that inversions form at different rates, or at different sizes, near the replication terminus.

Lastly, inversions may form with equal likelihood across the chromosome arm, but could be counter-selected near the replication terminus if operons there were longer, so that spontaneous inversions would be more likely to disrupt transcription units in that region. To test this hypothesis, we regressed operon length and number of genes per operon against distance of the operon from the terminus. There was no significant association with either metric in any of our 12 representative genomes (S6 Table). Therefore, we conclude that inversions would not disrupt transcription units to a greater degree near the replication terminus. Taken together, these analyses can find no relationship between the likelihood of inversion and distance from the replication terminus for any factor aside from the distribution of AIMS within bacterial genomes. Therefore, we conclude that these intragenomic rearrangements are counter-selected because they disrupt AIMS distributions.

### The distribution of inversions is not explained by *Ter* site abundance

Aside from AIMS, *Ter* sites in enteric bacteria are localized in proximity to the replication terminus [42–44]. *Ter* sites are longer and less abundant than AIMS, and serve to stall DNA polymerases travelling away from the replication terminus [45]. Inversion of individual *Ter* sites is highly detrimental as an inverted *Ter* site interrupts DNA replication before it is completed [46,47]. Analogous *Rtp* sites in *Bacillus* species also block retrograde replication and cannot be inverted [46–50]. Unlike highly abundant and nearly ubiquitous AIMS, *Ter* and *Rtp* sites are uncommon in the few genomes in which they are observed.

**Fig 4:**
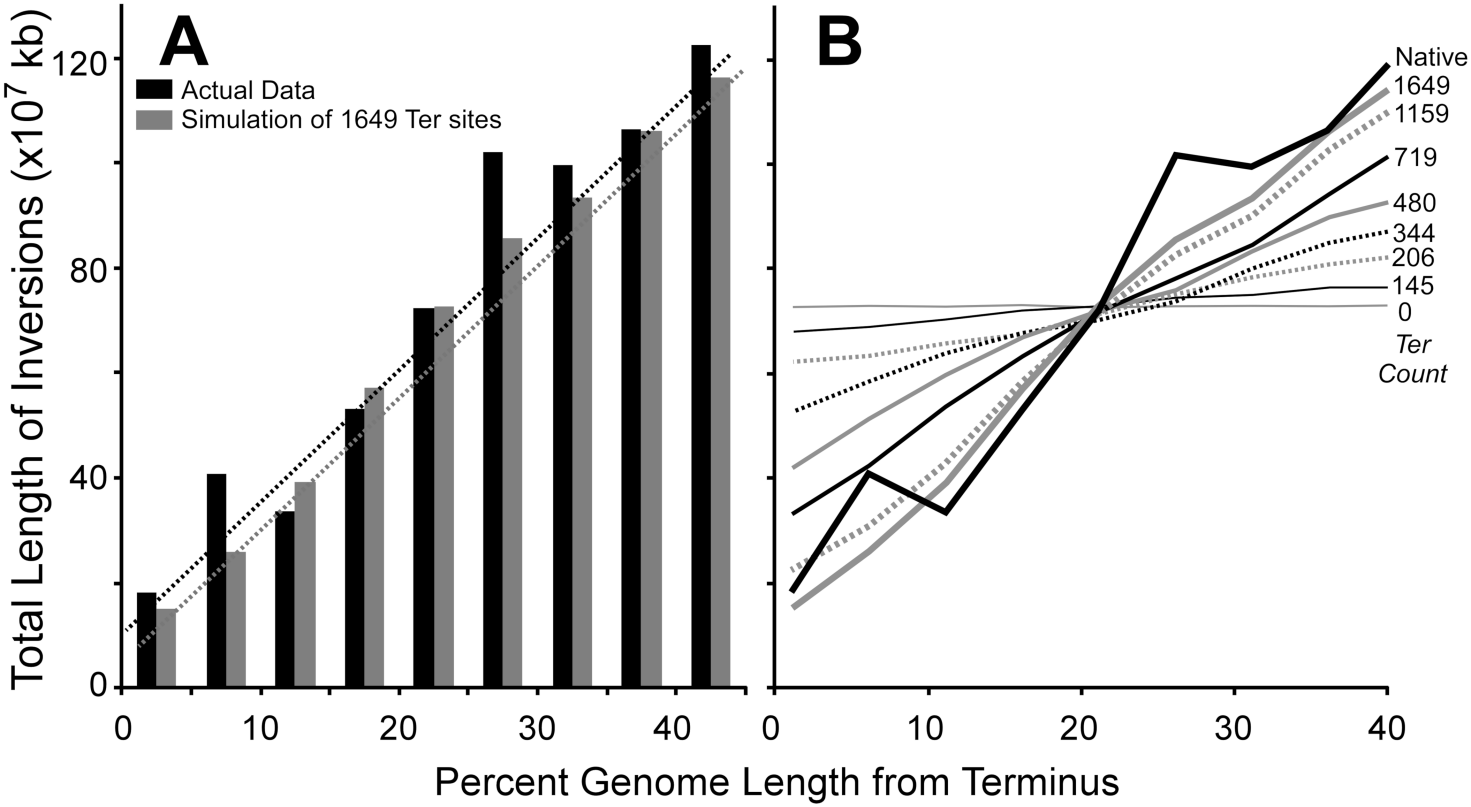
The frequency of *Ter* sites is insufficient to account for the inversion distribution. **A**. The distribution of inverted DNA with respect to the replication terminus in 213 genuine genomes (black bars) and simulated genomes (grey bars) with 1649 *Ter* sites placed in the genome. **B**. The distribution of inverted DNA in simulated genomes as a function of the number of simulated *Ter* sites placed in the genome (see Methods). The thick black line represents the distribution of inversions in genuine genomes.

To determine if the presence of known *Ter*-like sites could produce the distribution of inversions we observed, we simulated the random generation of inversions within a 4.5 MB genome that contained varying numbers of *Ter*-like sites placed in a gradient from replication origin to terminus. To simulate selection, simulated inversions containing a *Ter*-like site were considered nonpermissive and removed from the simulated data set. Each simulation was performed 100,000 times (Fig 4). For the actual number of *Ter* sites within the *E*. *coli* genome (<20), no impact on the distribution of inversions within chromosome arms was detected (Fig 4). To constrain inversions to the degree observed in genuine data, simulated genomes required ~1600 *Ter*-like sites to be placed in a positional gradient on each chromosome arm (~3200 per genome). This abundance of *Ter*-like sites is not consistent with the abundance of known *Ter* or *Rtp* sites, but is consistent with the abundance of AIMS. Therefore, we conclude that known low-abundance *Ter*-like sites could not have produced the distribution of inversions we observed.

### Observed HGT in completely sequenced genomes is compatible with AIMS structure

Just as AIMS distributions counter-select intragenomic rearrangements, we predict that intergenomic rearrangements that disrupt AIMS distributions will also be counter-selected. Upon insertion, newly-arrived DNA will contain AIMS in permissive and nonpermissive orientations at approximately equal frequencies. Inserted DNA should see minimal selection for AIMS function near the replication origin (Fig 1), so that acquired regions will show little strand-bias for AIMS. Selection for AIMS function increases with proximity to the replication terminus (Fig 1); therefore, we expect insertions which introduce AIMS in nonpermissive orientations to be removed more aggressively with proximity to the terminus. As a result, insertions in this region should bear AIMS in predominantly permissive orientations as seen, for example, in the abundance of KOPS (a subclass of AIMS) in prophages in *Salmonella* and *E*. *coli* [51,52].

**Fig 5:**
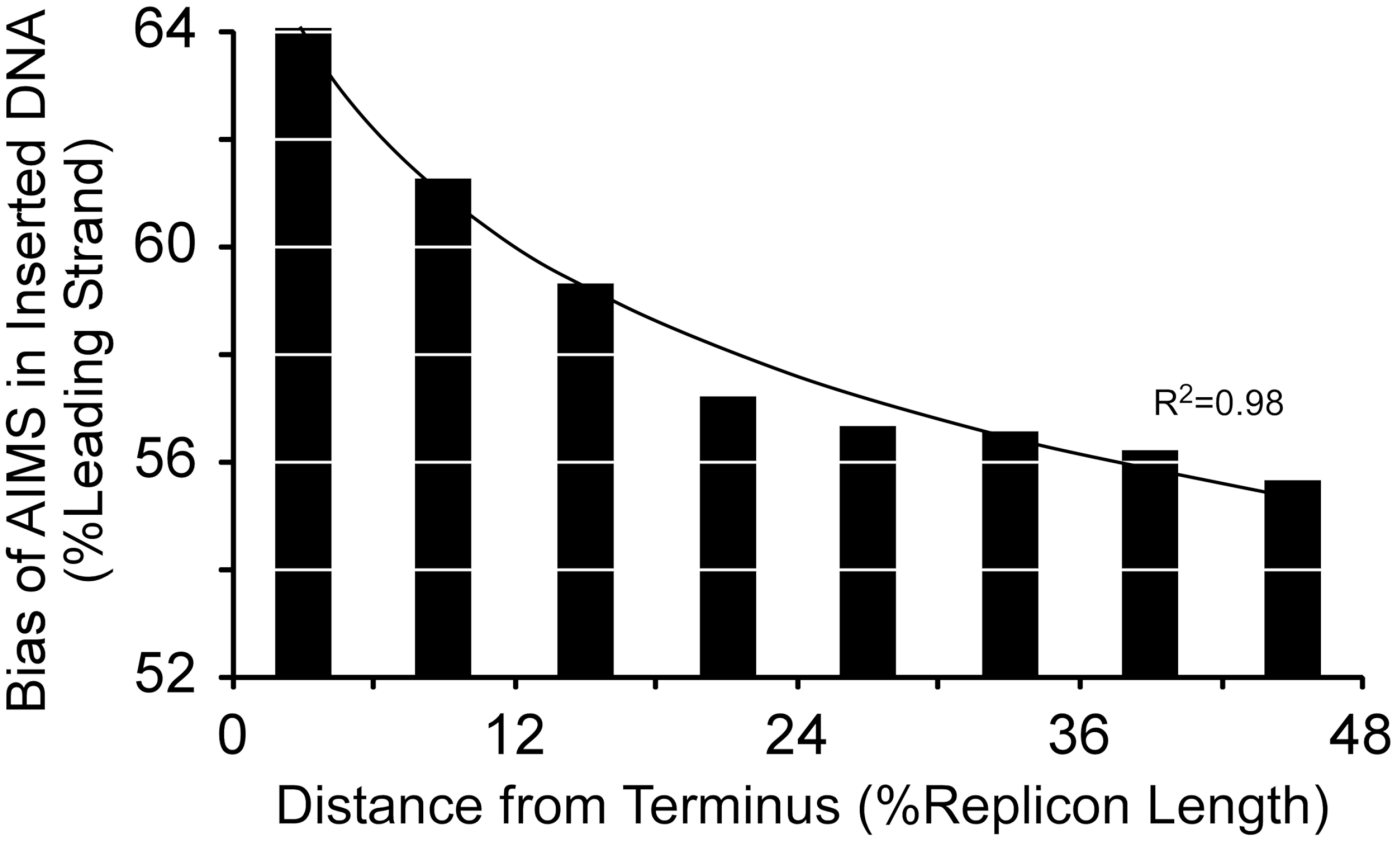
Strand-bias of AIMS in recently acquired genes as a function of the distance of the inserted DNA from the terminus. The data were fit to a negative exponential distribution.

To test this hypothesis, we identified 17,096 insertions totalling 36,434,039 bp of transferred DNA in 177 completely sequenced bacterial genomes (recipients) (S3 and S4 Tables). As described above, AIMS were identified in recipient genomes which lacked these insertions; that is, AIMS were identified in the backbone genome without considering their distribution in newly-acquired genes. We then enumerated the AIMS in permissive and nonpermissive orientations within each newly-acquired region. The strand-bias of AIMS within acquired regions was plotted against distance of the region from the replication terminus for all insertions in our dataset (Fig 5). Two conclusions can be drawn from these data. First, AIMS are strand-biased within DNA regions acquired by gene transfer even in the origin-proximal region of the chromosome. Second, a strong correlation was observed (R^2^ = 0.98), whereby the strand-bias of AIMS increased for insertions located near the replication terminus. If the analysis is limited to inserted regions up to 8 kb in length, the same pattern is observed (S2 Fig). Therefore, it is extremely unlikely that this pattern reflects the analysis of regions of native DNA that have been misannotated as “genes” and thus not identified in sibling strains or sister species.

As was true for the distribution of inversions above, this pattern was evident regardless of the taxonomy, genome size or nucleotide composition of the recipient genome (S3 Fig). We do not believe this reflects a process whereby DNA with permissive AIMS preferentially inserts near the terminus; rather, we surmise that insertions bearing nonpermissive AIMS have been counter-selected, and thus are observed less frequently, in the terminus region. The selection for AIMS function near the replication origin, while weaker than selection near the terminus, was still sufficient to counter-select fragments bearing AIMS in predominantly nonpermissive orientations, thus increasing average strand-bias of AIMS even in this location.

If the AIMS within inserted DNA arose from mutational processes after those genes’ acquisitions, then the strand-bias of AIMS within inserted DNA should increase with the length of time those sequences have dwelled in their recipient genomes. We used the average Ks between the most closely-related genomes bearing vs. lacking the insertion as a surrogate measure for the age of the insertion. We found that the increase of strand-bias of AIMS within terminus-proximal insertions is not a function of the average age of the insertion (S3 Fig); therefore, we conclude that the increase of strand-bias of AIMS towards the replication terminus does not reflect the action of mutation following the introduction of the foreign DNA.

### Constraints imposed by AIMS removes the majority of horizontally-acquired DNA

**Fig 6:**
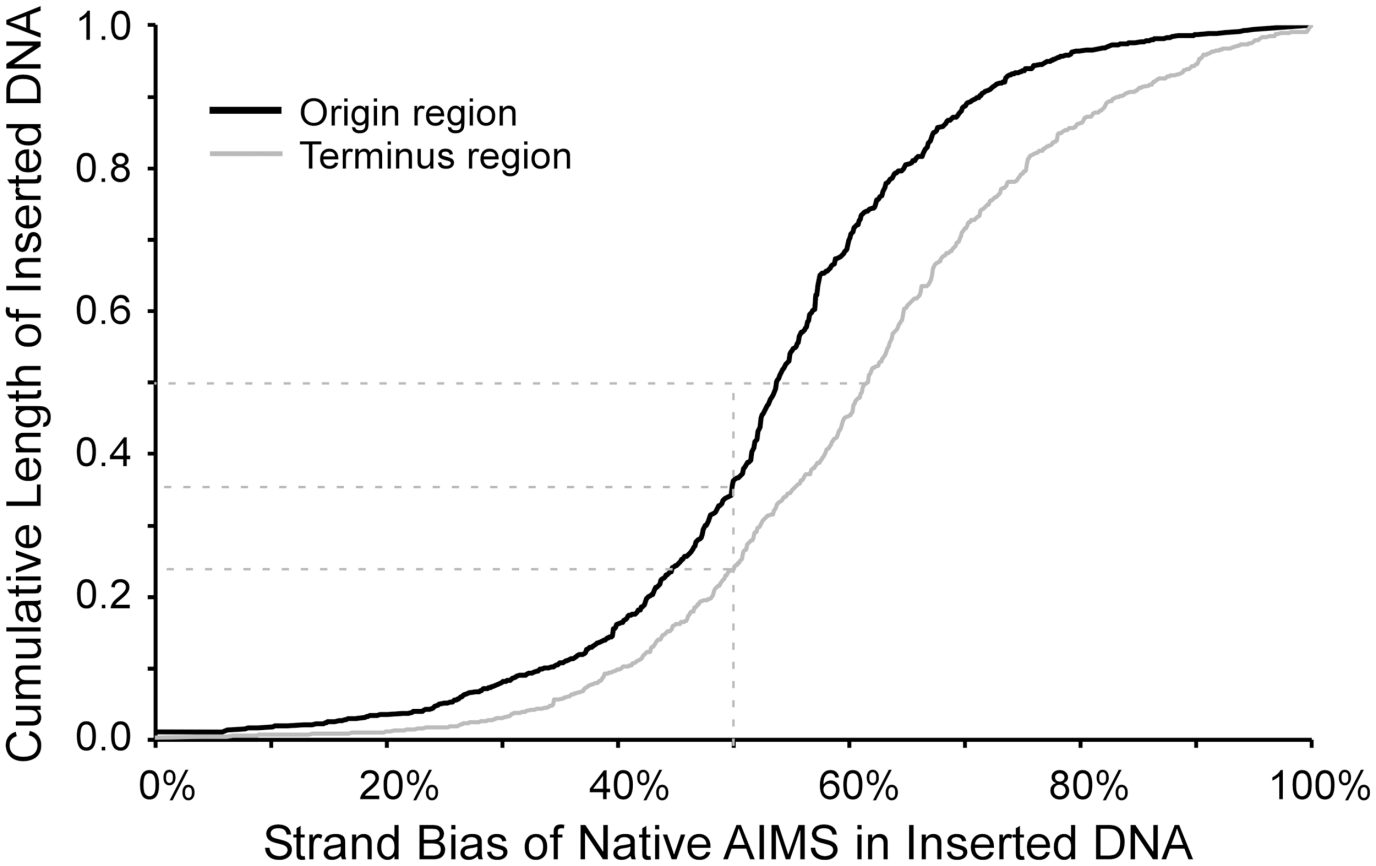
Loss of foreign DNA inserted in the terminus regions of genomes. A total of 10,707 insertions were catalogued in the genomes of γ-Proteobacteria; of these, 1597 insertions were located in the terminus-proximal 6% of the genomes, whereas 1220 insertions were located in the terminus-distal 6% of the genome (between 42 and 48% of the genome length from the terminus; the final 2% was ignored to accommodate differences in the lengths of chromosome arms). The cumulative length of the inserted fragments in each of these two regions is plotted against the strand-bias of native AIMS within each acquired fragment; as expected from Fig 5, DNA inserted near the replication terminus bear AIMS that are more strand-biased than fragments inserted near the replication origin. The shift of the strand-bias of AIMS in fragments inserted in the terminus region indicates a loss of 18% of the inserted fragments in this region.

To estimate the fraction of insertions that were removed due to selection for AIMS function, we analyzed genomes of γ-proteobacteria; the *dif* site locations in these taxa were most reliable, so that AIMS strand-bias on insertions near the terminus was most accurate. We compared insertions in the terminus region, where selection for AIMS function is expected to be strongest, to insertions in the origin region, where selection is weakest. For each region, the normalized cumulative length of the fragments was plotted, ordering fragments by the strand-bias of the native-genome’s AIMS within the fragment (Fig 6). In both chromosomal regions, acquired fragments bore AIMS predominantly in the permissive orientation; this is evident from the paucity of fragments with AIMS strand-bias less than 50%. As expected, the strand-bias of AIMS in fragments inserted near replication termini is even more pronounced (Figs 5 and 6, gray curve), differing significantly from the distribution of strand bias within origin-proximal fragments (P < 10^−16^, Kolmogorov-Smirnov test).

Using this cumulative distribution curve, we can estimate the fraction of fragments in the terminus region, relative to the origin region, that have been lost due to selection for AIMS function; this is accomplished by subtracting the areas under the normalized cumulative distribution curves. This analysis shows that at least 17.4% of fragments inserted near the replication terminus, relative to the replication origin, have been removed due to selection for AIMS function. This is, of course, an underestimate of the fraction of insertions lost due to selection for AIMS function because (a) the sets of fragments analyzed include very large numbers of genes that are recently acquired and have not yet been subject to selection [at least 90% of identified insertions[53], and (b) the absence of fragments with AIMS below 50% in the origin region indicates that selection for AIMS function has led to loss of fragments in the origin region as well. Even so, it demonstrates that selection for AIMS function imposes a significant and measurable barrier to the long-term persistence of inserted DNA in bacterial genomes.

### AIMS will restrict gene flow between higher taxonomic groups

**Fig 7:**
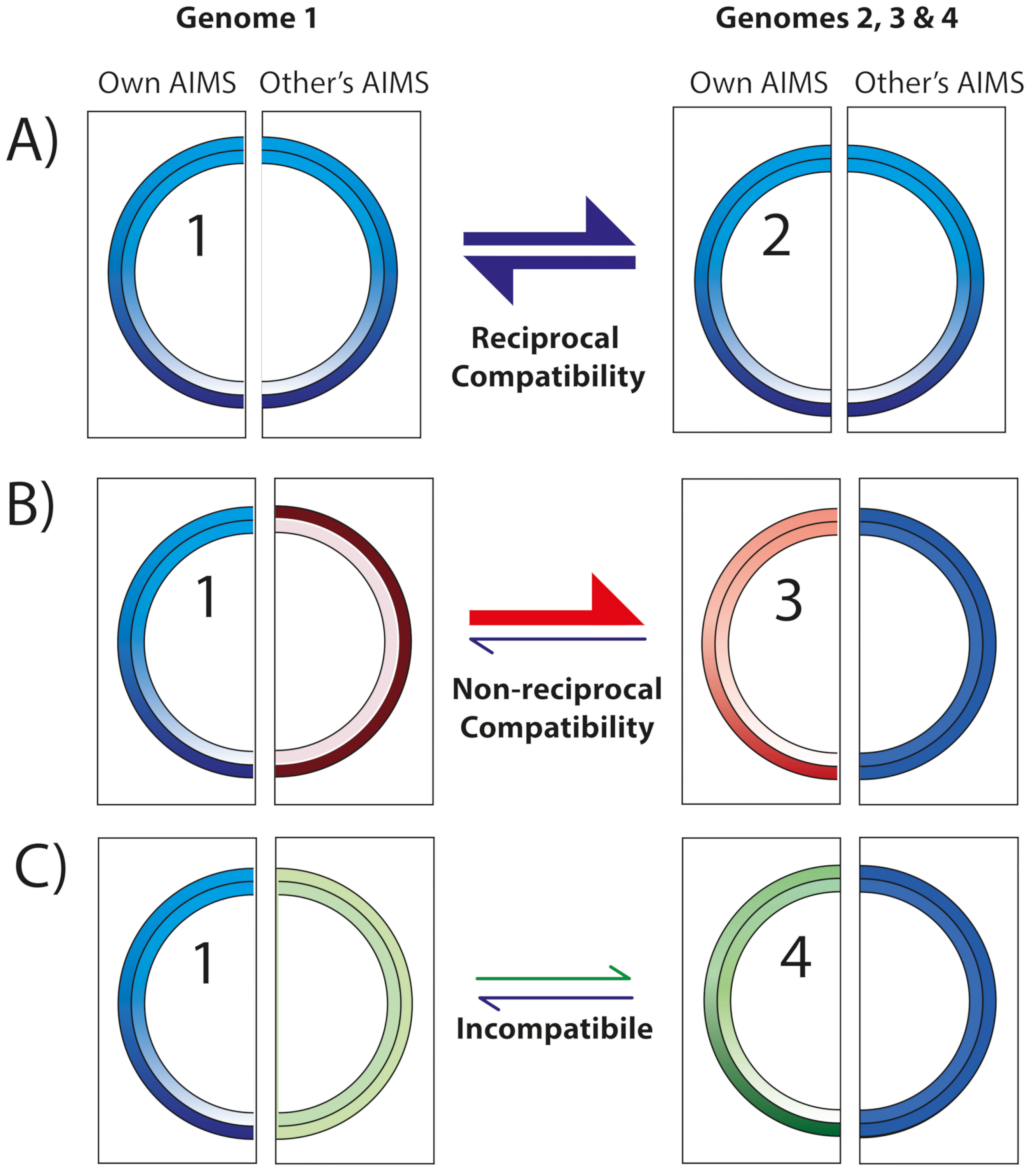
A model for the compatibility of genomes for gene transfer as a function of AIMS. AIMS in different compatibility groups are shown in different colors; darker colors indicate more abundant sequences. Genomes are numbers 1 through 4; colored bands indicate the abundance of oligomers that are AIMS within that genome (own AIMS) or AIMS within the partner genome (other’s AIMS). **A**. Genomes 1 and 2 share AIMS (blue); therefore, AIMS would not reduce gene transfer between these genomes. **B**. Gene transfer from genome 3 to genome 1 is reduced because sequences which serve as AIMS in genome 1 (blue) are not strand-biased in genome 3. However, gene transfer from genome 1 to genome 3 is not reduced because sequences serving as AIMS in genome 3 are strand-biased in genome 1 (red). **C**. AIMS reduces transfer between genomes 1 and 4 in both directions as sequences serving as AIMS in one genome (blue in genome 1, green in genome 4) are not strand-biased in the other genome.

Because AIMS provide a mechanism by which gene acquisition is constrained, they may act to bias overall gene flow between organisms of different taxonomic groups. Genomes will be more likely to acquire novel genes from donor taxa wherein the recipient genome’s AIMS are strand-biased (Figs 5 and 6). We posit that sets of AIMS found in any individual genome will be more likely to be strand-biased in genomes of related taxa, *e*.*g*., taxa in the same family or division; AIMS in the recipient taxon likely evolved function from simple strand-biased oligomers that were present in the common ancestor of both donor and recipient genomes. If so, then gene exchange would be more permissible between members of the same taxonomic group, and be more constrained between members of different taxonomic groups (Fig 7). Genomes would be more compatible for transfer if AIMS in a recipient genome are strand-biased in a donor genome.

**Fig 8:**
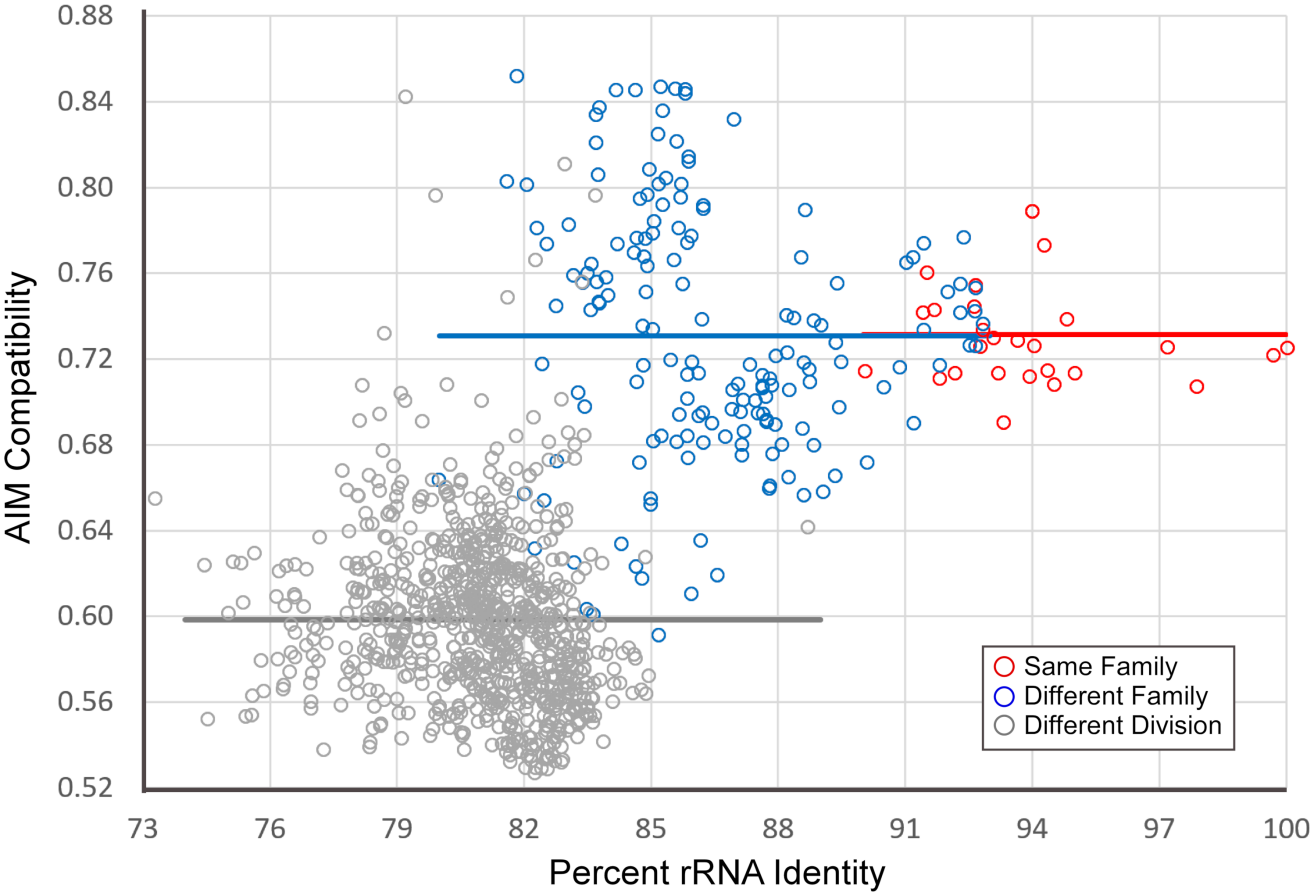
Compatibility of sets of AIMS within potential donor genomes to the AIMS found in the genome of *Bacillus subtilis* 168. The strand-bias of AIMS native to *B*. *subtilis* is plotted against the 16S rRNA identity between potential donor and recipient genomes. Lower values indicate that the AIMS in the recipient genome are less strand-biased in the donor genome. The red markers indicate donor genomes in the same family, the blue markers indicate donors in same division, but different family, and the gray markers indicate donors in different divisions. The red lines indicate least-squares linear regressions. Horizontal lines indicate mean compatibilities within each of these three groups.

To test this hypothesis, we identified AIMS in 119 taxa representing at least 54 families (some families were unknown) in 12 divisions; these were designated as potential recipient genomes. We then examined the degree of strand-bias for each set of AIMS within 1146 potential donor genomes, including taxa both closely- and distantly-related to each potential recipient genome. For each donor genome, the average strand-bias of oligomers which acted as AIMS in the 119 recipient taxa was assessed for 10 kb segments. Fig 8 shows representative data for the *Escherichia coli* and *Bacillus subtilis* genomes acting as potential recipients. In each case, the recipient genome’s AIMS were more strand-biased within more closely-related potential donor genomes (Fig 8). For recipients in each of the twelve divisions analyzed, donors from the same division were more compatible than donors in different divisions, and donors from the same family were always more compatible than donors from different families in the same division (Table 1). Because successful HGT events are more likely to involve donor genomes with compatible AIMS (Fig 7), these data support the hypothesis that AIMS will counter-select HGT events from more distantly-related taxa. Thus, these data suggest that donor taxa from the same division (or family) would introduce DNA fragments with AIMS in the permissive orientations more often than donor taxa from different divisions (or families).

**Table 1:** Average bias of AIMS within donor fragments.

Division	Same Division		Different Division			Same Division Same Family		Same Division Different Family		
	N^1^	Bias^2^	N	Bias	Delta^3^	N	Bias	N	Bias	Delta^3^
Actinobacteria	486	57.36	4527	56.94	0.42	42	59.33	444	57.17	2.16
Alphaproteobacteria	660	56.74	4910	56.87	-0.13	51	58.10	609	56.63	1.47
Bacteroidetes	54	56.31	1617	56.09	0.22	8	62.45	46	55.25	7.21
Betaproteobacteria	185	57.88	2600	57.67	0.21	26	59.70	159	57.58	2.13
Chlamydiae	12	60.76	1102	55.61	5.15	10	62.04	2	54.33	7.71
Cyanobacteria	69	54.34	1602	55.19	-0.85	4	57.71	65	54.13	3.58
Deltaproteobacteria	81	56.86	1590	56.27	0.59	17	61.10	64	55.73	5.37
Epsilonproteobacteria	30	57.78	1084	56.93	0.86	12	59.19	18	56.84	2.35
Firmicutes	1344	65.50	7568	56.12	9.38	167	66.65	1177	65.34	1.31
Gammaproteobacteria	2369	58.54	10442	57.46	1.07	284	59.98	2085	58.34	1.65
Spirochaetes	24	64.80	1647	57.85	6.95	12	68.40	12	61.21	7.19
Tenericutes	51	58.21	1620	54.76	3.45	28	59.20	23	56.99	2.21

^1^. Number of comparisons averaged.

^2^. Average strand-bias of recipient AIMS in donor genomes.

^3^. The difference between the same/difference division biases, or the same/different family biases.

### Conclusion

Horizontal gene transfer is a powerful source of genetic and physiological change in bacteria. It has been suggested that genotypic and phenotypic cohesion is observed at higher taxonomic levels in bacteria despite rampant HGT [4,10,54]. While Gogarten *et al*. [9] proposed that this cohesion could reflect barriers to HGT between organisms in different higher taxonomic groups, no mechanisms had been identified. Here, we propose that any benefit conferred by an introduced gene must offset any detriment incurred by its integration into the genome; such detriments would arise if the incoming DNA fragment contained AIMS in preferentially non-permissive orientations. Our data demonstrate that AIMS likely constrain both intragenomic and intergenomic rearrangements, that substantial numbers of introduced genes were eliminated due to their failure to have AIMS in the permissive orientations, and that genomes within higher taxonomic groups are more compatible for gene transfer than genomes outside those groups due to donor genomes bearing recipient genomes’ AIMS as strand-biased oligomers. Thus, selection for the preservation of AIMS-based genome architecture provides a much-needed mechanism for the preferential transfer of genes among organisms of higher taxonomic groups. This, in turn, provides a mechanism whereby genotypic and phenotypic similarities among taxa within higher taxonomic groups do not reflect ancestral characteristics, but rather more frequent gene exchange.

## Materials & methods

### Genomes, sequences and software

All genome sequences were retrieved from GenBank; genes were defined using the annotations provided. Orthologues in strains of the same species were identified as reciprocal best BLAST hits where (a) encoded proteins exceeded 70% similarity or encoded structural RNAs exceeded 90% identity, and (b) >85% of coding sequences were aligned. A consensus sequence of 5’-RNTKCGCATAATGTATATTATGTTAAAT was used to locate putative *dif* sites in γ-proteobacterial genomes. A consensus sequence of 5’- AGNATGTTGTAACTAA was used to locate Ter sites in the *E*. *coli* genome. All analyses were performed using DNA Master version 5.23, available from cobamide2.bio.pitt.edu.

### Identifying the replication origin and terminus

The replication origins and termini were identified using the relative abundance of strand-biased pentamers. Possible intergenic locations of the replication origin and terminus were permuted across the genome, creating two potential chromosome arms. The relative frequency of pentamers was quantified within each of the three reading frames of protein-coding genes as,

$$f_{ijklm,r}=\sum_{r}\sum_{i}\sum_{j}\sum_{k}\sum_{l}\sum_{m}P\left(B_{m}|T_{ijkl}\right)$$ (1)

where *r* is the reading frame, *ijklm* are five consecutive nucleotide positions, *T* is the specific tetramer at position *ijkl*, *B*_*m*_ is the identity of the base at position *m*, and P(*B*|*T*) is the probability of base *B* given tetramer *T*. Values are summed across all 3 reading frames and all 4 nucleotides. The difference in pentamer frequencies Δ was calculated as the sum of the squared differences between genes on putative leading *vs*. lagging strands:

$$\Delta =\sum_{r}\sum_{i}\sum_{j}\sum_{k}\sum_{l}\sum_{m}{\left(f_{ijklm,r,Lead}-f_{ijklm,r,Lag}\right)}^{2}$$ (2)

The replication breakpoints were identified as those locations that maximized Δ, the differences in relative, frame-specific pentamer frequencies between genes predicted to be transcribed on leading *vs*. lagging strands. The two breakpoints were assigned as the replication origin or terminus so that the number of genes transcribed away from the replication origin was maximized. The positions of the termini were validated using the locations of known *dif* sites, which are found at replication termini [30]. This validation also demonstrated that replication breakpoints identified using pentamer distributions were more robust than those identified using GC skew. The final dataset used only genomes with curated *dif* sites [55,56], further substantiating the origins identified using the method described.

### Identifying arm-specific inversions

Inversions were identified in organisms with at least 97% 16S rRNA similarity; inversions were evident within a backbone of syntenic genes as regions where gene orientations were reversed relative to adjacent genes. Using uppercase and lowercase letters to represent genes transcribed from the leading and lagging strands, respectively, genes DEF would be inverted if region ABCDEFGHJ were organized as ABCfedGHJ in a sister taxon. We ignored potential rearrangements where flanking genes lacked synteny and thus may represent translocations or xenologous insertions. Inversions including the replication origin or terminus were ignored as these do not invert AIMS. The midpoint of each inversion was used to calculate distance from the terminus, normalized as a percentage of the total genome length and averaged between the two genomes. In identifying inversions among multiple taxa, inversion identified in multiple comparisons were counted only once.

### Identifying genes gained by horizontal gene transfer

Genes likely to have been acquired by horizontal gene transfer were identified as those lacking an orthologue in the genomes of a sister species as well as multiple strains of the same species, where the closest homologue in a conspecific strain encoded a protein with < 40% similarity. The absence of the gene in multiple strains increases the likelihood that the gene was a novel acquisition rather than a parallel loss. The location of the insertion was quantified as the percentage of the genome length of the midpoint of the insertion from the replication terminus.

### Identifying AIMS

AIMS were identified in genomes in which horizontally transferred genes had been identified and removed from the sequence as above. AIMS were identified as 8-mer sequences with increased abundance, as well as increased strand-bias, in the 25% of the genome near the replication terminus relative to the values observed for the 60% of the genome near the replication origin [16]. Degenerate octamers are useful surrogates for detecting selection on longer sequences whose direct detection is not robust; longer sequences are generally too infrequent to allow reliable measures of changes in abundance across the chromosome. The thresholds for increase in skew and abundance were established for each genome such that the number of observed AIMS in genuine genomes exceeded the numbers identified in resampled genomes by at least 10-fold. Resampled genomes were constructed by randomly rearranging 40 kb segments within each chromosome arm, thus preserving leading and lagging strand-bias. Sets of AIMS included those that (a) were highly abundant, but had weaker increase in strand-bias near the terminus, and (b) were less abundant but with strong increase in strand-bias near the terminus. The final sets of AIMS used herein are outlined in S6 Table.

### Simulated *Ter* distributions

To examine the number of *Ter* sites required to decrease the occurrence of inversions near the replication terminus, simulated *Ter* sites were inserted in a simulated genome where inter-*Ter* distance increased linearly with distance from the terminus. Simulated inversions were then generated at random within the genome, where the distribution of inversion size was modelled after those seen in genuine data; simulated inversions were discarded (counter-selected) if they included a simulated *Ter* site.

### AIMS compatibility

To determine the compatibility for gene exchange between genomes, we measured the strand-bias of a recipient genome’s AIMS within a donor genome. Biases were measured within randomly chosen 10 kb segments of potential donor genomes; this method allows us to determine the AIMS composition of DNA fragments in a donor genome without the need to predict its replication origin or terminus. Instances of each of the recipient genome’s AIMS were counted on the Watson (N_W_) and Crick (N_C_) strands of each donor DNA fragment; the strand-bias of each AIMS (SB_i_) was calculated as

$${\text{SB}}_{\text{i}}={\text{Supremum}\;\text{of}\;\text{N}}_{\text{W}}/\left({\text{N}}_{\text{W}}+{\text{N}}_{\text{C}}\right){\;\text{and}\;\text{N}}_{\text{C}}/\left({\text{N}}_{\text{W}}+{\text{N}}_{\text{C}}\right).$$ (3)

The mean strand-bias of recipient AIMS in a donor genome ($\bar{{SB}_{i}}$) was calculated as the mean strand-bias for 1000 randomly chosen 10 kb donor fragments. The overall compatibility between genomes X and Y (C_XY_) was calculated as

$$C_{XY}=\sum_{i}\bar{SB_{i}}*N_{i}/\sum_{i}N_{i}$$ (4)

where N_i_ is the abundance of AIMS *i* in the recipient genome. Values are summed across all AIMS in the recipient genomes. Thus, compatibility represents a mean bias of a recipient genome’s AIMS in the donor genome, weighted for the abundance of the AIMS in the recipient genome. We do not weight the contributions of individual AIMS by their strand bias in the recipient genome since this is a function of both selection and mutational bias.

## Supporting information

S1 Dataset Sets of AIMS identified in bacterial genomes. (XLSX)

S1 Table Phylogenetic distributions of sources of 634 inversions. (PDF)

S2 Table Comparisons used to identify 634 inversions. (PDF)

S3 Table Phylogenetic distributions of sources of 17096 insertions. (PDF)

S4 Table Genomes used to identify 17096 insertions. (PDF)

S5 Table Predicted replication breakpoints. (PDF)

S6 Table Correlation of gene data with distance from the replication terminus. (PDF)

S7 Table Average bias of AIMS within donor fragments. (PDF)

S1 Fig Distribution of inversions in completely sequenced bacterial genomes. A total of 634 inversions were identified in 159 pairwise comparisons of 214 separate completely sequenced genomes (See S2 Table for details). All data are plotted as % genome distance of the midpoint of the inversion from the replication terminus. The total length of DNA inverted plotted by genome position across all genomes included in the analysis.
(PDF)

S2 Fig Strand bias of AIMS in recently acquired genes filtered by minimum size for inserted region. Strand-bias is assessed for insertions with within chromosomal regions with increasing distance from the replication terminus. Black bars depict average strand bias for all genes (data also presented in Fig 5). Gray bars depict average strand bias for subsets of data whereby the clusters of contiguous inserted genes analysed must lie in regions larger than 1kb, 2 kb, 4 kb or 8 kb.
(PDF)

S3 Fig Strand bias of AIMS in recently acquired genes. Strand-bias is assessed for insertions with within chromosomal regions with increasing distance from the replication terminus. **A**. Organisms are segregated into γ-Proteobacteria and other divisions; other divisions lack the sample size to assay individually. **B**. Organisms are segregated by GC content. **C**. Organisms are segregated by genome size. **D**. Organisms are segregated by the average divergence at synonymous sites between the organisms bearing the insertion and the most closely-related genome which lacks the insertion, thus placing an upper bound on the age of the insertion within the recipient genome.
(PDF)
