# Supplementary material for: Chromosome architecture constrains horizontal gene transfer in bacteria
Source: PLoS Genet. 2018 May 29;14(5):e1007421. doi: 10.1371/journal.pgen.1007421 (PMC5993296; doi:10.1371/journal.pgen.1007421)
Supplement: S1 Table — (PDF) [file pgen.1007421.s002.pdf]

Table S1. Phylogenetic distribution of sources of 634 inversions

Division	Family	Inversions
**Actinobacteria**		**88**
	Bifidobacteriaceae	4
	Corynebacteriaceae	13
	Frankiaceae	12
	Microbacteriaceae	3
	Micrococcaceae	12
	Micromonosporaceae	5
	Mycobacteriaceae	19
	Nocardiaceae	10
	Streptomycetaceae	10
**α-Proteobacteria**		**205**
	Anaplasmataceae	5
	Bartonellaceae	19
	Bradyrhizobiaceae	36
	Caulobacteraceae	15
	Rhizobiaceae	111
	Rickettsiaceae	8
	Sphingomonadaceae	11
**Bacteroidetes**	**Bacteroidaceae**	**17**
**β-Proteobacteria**	**Burkholderiaceae**	**19**
**Chlamydiae**	**Chlamydiaceae**	**14**
**Chlorobi**	**Chlorobiaceae**	**10**
**Chloroflexi**		**14**
	Chloroflexaceae	11
	Unknown	3
**Cyanobacteria**	**Prochlorococcaceae**	**28**
**Deinococcus-Thermus**	**Thermaceae**	**1**
**δ-Proteobacteria**		**17**
	Geobacteraceae	1
	Myxococcaceae	16
**Dictyoglomi**	**Dictyoglomaceae**	**2**
**ε-Proteobacteria**	**Helicobacteraceae**	**16**
**Firmicutes**		**95**
	Bacillaceae	34
	Clostridiaceae	14
	Lactobacillaceae	18
	Listeriaceae	1
	Peptococcaceae	7
	Streptococcaceae	7
	Thermoanaerobacteraceae	5
	Unknown	9
**γ-Proteobacteria**		**92**
	Enterobacteriaceae	89
	Vibrionaceae	3
**Spirochaetes**	**Leptospiraceae**	**5**
**Tenericutes**	**Mycoplasmataceae**	**5**
**Thermotogae**	**Thermotogaceae**	**6**
**Total**		**634**
