# Supplementary material for: Chromosome architecture constrains horizontal gene transfer in bacteria
Source: PLoS Genet. 2018 May 29;14(5):e1007421. doi: 10.1371/journal.pgen.1007421 (PMC5993296; doi:10.1371/journal.pgen.1007421)
Supplement: S2 Table — (PDF) [file pgen.1007421.s003.pdf]

Table S2. Comparisons used to identify 634 inversions

Organism	Accession	Organism	Accession	Inversion Count	Total Features	Total Length (kb)
Arthrobacter aurescens TC1	NC_008711	*Arthrobacter chlorophenolicus* A6	NC_011886	10	15	5421.8
Arthrobacter aurescens TC1	NC_008711	*Arthrobacter* sp. FB24	NC_008541	2	2	822.0
Bifidobacterium longum	NC_010816	*Bifidobacterium longum* NCC2705	NC_004307	3	4	1498.0
Bifidobacterium longum	NC_010816	*Bifidobacterium longum* ATCC 15697	NC_011593	1	1	25.7
Clavibacter michiganensis NCPPB 382	NC_009480	*Clavibacter michiganensis*	NC_010407	3	79	24648.0
Corynebacterium aurimucosum	NC_012590	*Corynebacterium efficiens* YS-314	NC_004369	4	12.5	3154.5
Corynebacterium aurimucosum	NC_012590	*Corynebacterium kroppenstedtii* DSM 44385	NC_012704	9	16	2347.5
Frankia alni ACN14a	NC_008278	*Frankia* sp. CcI3	NC_007777	12	36	13351.5
Mycobacterium smegmatis JS623	CP003078	*Mycobacterium smegmatis* MC^2 155	NC_008596	9	22	7483.7
Mycobacterium tuberculosis CDC1551	NC_002755	*Mycobacterium leprae* TN	NC_002677	5	28.5	10999.3
Mycobacterium tuberculosis CDC1551	NC_002755	*Mycobacterium smegmatis* MC^2 155	NC_008596	3	37	11791.2
Rhodococcus jostii RHA1	NC_008268	*Rhodococcus opacus* B4	NC_012522	4	6.5	2487.7
Rhodococcus jostii RHA1	NC_008268	*Rhodococcus erythropolis* PR4	NC_012490	6	20.5	6677.3
Salinispora arenicola CNS-205	NC_009953	*Salinispora tropica* CNB-440	NC_009380	5	48.5	15073.3
Streptomyces coelicolor A3(2)	NC_003888	*Streptomyces griseus* NBRC 13350	NC_010572	1	5	1811.7
Streptomyces coelicolor A3(2)	NC_003888	*Streptomyces scabiei* 87.22	NC_013929	5	8.5	3335.7
Agrobacterium radiobacter K84	NC_011985	*Agrobacterium tumefaciens* C58	NC_003062	15	127	39934.3
Agrobacterium radiobacter K84	NC_011985	*Agrobacterium vitis* S4	NC_011989	19	310.5	107286.7
Agrobacterium radiobacter K84	NC_011985	*Rhizobium* NGR234	NC_012587	19	182.5	60289.0
Agrobacterium radiobacter K84	NC_011985	*Rhizobium etli* CFN 42	NC_007761	11	137.5	55647.0
Agrobacterium radiobacter K84	NC_011985	*Rhizobium etli* CIAT 652	NC_010994	4	26	7659.3
Agrobacterium radiobacter K84	NC_011985	*Rhizobium leguminosarum* WSM1325	NC_012850	4	24	6299.8
Agrobacterium radiobacter K84	NC_011985	*Rhizobium leguminosarum* WSM2304	NC_011369	3	7.5	1450.0
Agrobacterium radiobacter K84	NC_011985	*Rhizobium leguminosarum* 3841	NC_008380	3	7.5	1431.3
Agrobacterium radiobacter K84	NC_011985	*Sinorhizobium medicae* WSM419	NC_009636	8	103.5	35232.2
Agrobacterium radiobacter K84	NC_011985	*Sinorhizobium meliloti* 1021	NC_003047	7	120	40901.8
Anaplasma marginale Florida	NC_012026	*Anaplasma marginale* St. Maries	NC_004842	5	13	3963.8
Bartonella tribocorum CIP 105476	NC_010161	*Bartonella grahamii* as4aup	NC_012846	1	1	340.5
Bartonella tribocorum CIP 105476	NC_010161	*Bartonella australis* Aust/NH1	CP003123	8	40	14270.5
Bartonella tribocorum CIP 105476	NC_010161	*Bartonella bacilliformis* KC583	NC_008783	2	10.5	3647.5
Bartonella tribocorum CIP 105476	NC_010161	*Bartonella clarridgeiae* 73	FN645454	3	21	8038.2
Bartonella tribocorum CIP 105476	NC_010161	*Bartonella henselae* Houston-1	NC_005956	2	2	860.0
Bartonella tribocorum CIP 105476	NC_010161	*Bartonella quintana* RM-11	CP003784	2	3	863.8
Bartonella tribocorum CIP 105476	NC_010161	*Bartonella vinsonii* Winnie	CP003124	1	9	3783.0
Buchnera aphidicola APS	NC_002528	*Buchnera aphidicola* Bp	NC_004545	2	6	2136.2
Buchnera aphidicola APS	NC_004545	*Buchnera aphidicola* Cc	NC_008513	1	2	958.3
Buchnera aphidicola APS	NC_004545	*Buchnera aphidicola* Sg	NC_004061	1	3.5	1542.2
Caulobacter crescentus NA1000	NC_011916	*Caulobacter segnis* ATCC 21756	CP002008	8	104.5	35805.2
Caulobacter crescentus CB15	NC_002696	*Caulobacter segnis* ATCC 21756	CP002008	7	84.5	30106.5
Rickettsia akari Hartford	NC_009881	*Rickettsia canadensis* McKiel	NC_009879	3	79	27000.2
Rickettsia akari Hartford	NC_009881	*Rickettsia conorii* Malish 7	NC_003103	3	65	16247.8
Rickettsia akari Hartford	NC_009881	*Rickettsia felis* URRWXCal2	NC_007109	1	34	9210.0
Rickettsia akari Hartford	NC_009881	*Rickettsia prowazekii* Madrid E	NC_000963	1	45	14317.7
Sinorhizobium meliloti 1021	NC_003047	*Sinorhizobium fredii* HH103	HE616890	8	76	26298.8
Sinorhizobium fredii NGR234	CP001389	*Sinorhizobium fredii* HH103	HE616890	2	17.5	6883.7
Sinorhizobium fredii USDA 257	CP003563	*Sinorhizobium fredii* HH103	HE616890	8	92.5	28677.7
Zymomonas mobilis NCIMB 11163	NC_013355	*Zymomonas mobilis* ATCC 29191	CP003704	3	34	12175.8
Zymomonas mobilis NCIMB 11163	NC_013355	*Zymomonas mobilis* ATCC 29192	CP002865	6	103	37957.0
Zymomonas mobilis ZM4	NC_006526	*Zymomonas mobilis* ATCC 10988	CP002850	1	418.5	145982.2

Zymomonas mobilis ZM4	NC_006526	*Zymomonas mobilis* ATCC 29192	CP002865	1	1	25.2
Burkholderia ambifaria AMMD	NC_008390	*Burkholderia ambifaria* MC40-6	NC_010551	2	2	556.0
Burkholderia ambifaria AMMD	NC_008390	*Burkholderia mallei* ATCC 23344	NC_006348	4	106	39520.0
Burkholderia ambifaria AMMD	NC_008390	*Burkholderia mallei* NCTC 10229	NC_008836	2	34	12978.8
Burkholderia ambifaria AMMD	NC_008390	*Burkholderia phymatum* STM815	NC_010622	2	5	2228.2
Burkholderia cenocepacia HI2424	NC_008542	*Burkholderia mallei* ATCC 23344	NC_006348	4	96	36689.2
Burkholderia cenocepacia HI2424	NC_008542	*Burkholderia mallei* NCTC 10229	NC_008836	3	60.5	20529.3
Burkholderia cenocepacia HI2424	NC_008542	*Burkholderia phymatum* STM815	NC_010622	2	3	712.2
Chlamydia muridarum Nigg	NC_002620	*Chlamydophila abortus* S26/3	NC_004552	11	21.5	5693.7
Chlamydia muridarum Nigg	NC_002620	*Chlamydophila caviae* GPIC	NC_003361	1	1	161.5
Chlamydia muridarum Nigg	NC_002620	*Chlamydophila pneumoniae* AR39	NC_002179	2	2	103.7
Chlorobium phaeobacteroides BS1	NC_010831	*Prosthecochloris aestuarii* DSM 271	NC_011059	8	9	3088.8
Chlorobium phaeobacteroides BS1	NC_010831	*Chlorobium phaeovibrioides* DSM 265	NC_009337	2	2	407.5
Chloroflexus sp. Y-400-fl	NC_012032	*Chloroflexus aggregans* DSM 9485	NC_011831	11	269	95485.2
Dehalococcoides ethenogenes 195	NC_002936	*Dehalococcoides* sp. BAV1	NC_009455	1	5	1084.8
Dehalococcoides ethenogenes 195	NC_002936	*Dehalococcoides* VS	NC_013552	2	2	437.0
Prochlorococcus marinus MIT 9215	NC_009840	*Prochlorococcus marinus* MIT 9301	NC_009091	3	8.5	2566.2
Prochlorococcus marinus MIT 9215	NC_009840	*Prochlorococcus marinus* MIT 9303	NC_008820	2	111	31922.3
Prochlorococcus marinus MIT 9215	NC_009840	*Prochlorococcus marinus* MIT 9313	NC_005071	1	18	5966.2
Prochlorococcus marinus MIT 9215	NC_009840	*Prochlorococcus marinus* MIT 9515	NC_008817	6	6	1035.5
Prochlorococcus marinus MIT 9215	NC_009840	*Prochlorococcus marinus* CCMP1375	NC_005042	2	14	4926.7
Prochlorococcus marinus MIT 9215	NC_009840	*Prochlorococcus marinus* CCMP1986	NC_005072	2	2	490.5
Prochlorococcus marinus MIT 9312	NC_007577	*Prochlorococcus marinus* MIT 9313	NC_005071	2	34	11299.7
Prochlorococcus marinus MIT 9312	NC_007577	*Prochlorococcus marinus* NATL1A	NC_008819	2	3	974.3
Prochlorococcus marinus MIT 9312	NC_007577	*Prochlorococcus marinus* NATL2A	NC_007335	1	1	223.0
Prochlorococcus marinus MIT 9303	NC_008820	*Prochlorococcus marinus* MIT 9312	NC_007577	2	47.5	16966.8
Prochlorococcus marinus MIT 9211	NC_009976	*Prochlorococcus marinus* MIT 9312	NC_007577	1	2.5	589.7
Prochlorococcus marinus MIT 9215	NC_009840	*Prochlorococcus marinus* MIT 9303	NC_008820	2	46	16263.7
Prochlorococcus marinus MIT 9215	NC_009840	*Prochlorococcus marinus* MIT 9515	NC_008817	1	124	31752.2
Prochlorococcus marinus MIT 9312	NC_007577	*Prochlorococcus marinus* MIT 9515	NC_008817	1	1	235.0
Anaeromyxobacter dehalogenans 2CP 1	NC_011891	*Anaeromyxobacter* sp. K	NC_011145	3	14.5	4547.7
Anaeromyxobacter dehalogenans 2CP 1	NC_011891	*Anaeromyxobacter* sp. Fw109-5	NC_009675	9	32.5	12144.0
Anaeromyxobacter dehalogenans 2CP-C	NC_007760	*Anaeromyxobacter dehalogenans* 2CP 1	NC_011891	4	13	4814.7
Dictyoglomus thermophilum H-6-12	NC_011297	*Dictyoglomus turgidum* DSM 6724	NC_011661	2	3	114.3
Helicobacter pylori 26695	NC_000915	*Helicobacter pylori* HPAG1	NC_008086	3	17.5	5625.8
Helicobacter pylori 26695	NC_000915	*Helicobacter pylori* G27	NC_011333	2	9	3983.8
Helicobacter pylori 26695	NC_000915	*Helicobacter pylori* J99	NC_000921	4	15.5	6266.2
Helicobacter pylori 26695	NC_000915	*Helicobacter pylori* P12	NC_011498	1	1	142.0
Helicobacter pylori G27	NC_011333	*Helicobacter pylori* B38	NC_012973	1	2	819.0
Helicobacter pylori J99	NC_000921	*Helicobacter pylori* B38	NC_012973	1	1	275.0
Helicobacter pylori P12	NC_011498	*Helicobacter pylori* B38	NC_012973	2	23	8406.2
Helicobacter pylori Shi470	NC_010698	*Helicobacter pylori* B38	NC_012973	2	4	756.0
Alkaliphilus metalliredigens QYMF	NC_009633	*Alkaliphilus oremlandii* OhILAs	NC_009922	3	32.5	9260.8
Bacillus amyloliquefaciens FZB42	NC_009725	*Bacillus subtilis* 168	NC_000964	2	2	706.0
Bacillus pumilus SAFR-032	NC_009848	*Bacillus subtilis* 168	NC_000964	5	20	6264.2
Bacillus pumilus SAFR-032	NC_009848	*Bacillus licheniformis* ATCC 14580	NC_006322	9	24	8093.0
Bacillus clausii KSM-K16	NC_006582	*Bacillus halodurans* C-125	NC_002570	2	2	408.0
Bacillus pumilus SAFR-032	NC_009848	*Bacillus weihenstephanensis* KBAB4	NC_010184	6	8	2233.3
Bacillus pumilus SAFR-032	NC_009848	*Bacillus halodurans* C-125	NC_002570	4	5.5	1017.0
Clostridium botulinum A Hall	NC_009698	*Clostridium botulinum* A ATCC 3502	NC_009495	2	5	152.7
Clostridium botulinum A Hall	NC_009698	*Clostridium botulinum* A2 Kyoto	NC_012563	1	1	179.0
Clostridium botulinum A Hall	NC_009698	*Clostridium botulinum* Ba4 657	NC_012658	1	1	38.0

Clostridium kluyveri DSM 555	NC_009706	*Clostridium tetani* E88	NC_004557	6	10.5	3199.8
Clostridium kluyveri DSM 555	NC_009706	*Clostridium perfringens* str. 13	NC_003366	1	7.5	2237.0
Desulfitobacterium hafniense DCB-2	NC_011830	*Desulfitobacterium hafniense* Y51	NC_007907	1	1	527.0
Desulfotomaculum reducens MI-1	NC_009253	*Desulfotomaculum acetoxidans* DSM 771	NC_013216	6	9	1952.5
Exiguobacterium sibiricum 255-15	NC_010556	*Exiguobacterium* sp. AT1b	NC_012673	6	29	8815.7
Geobacillus kaustophilus HTA426	NC_006510	*Geobacillus thermodenitrificans* NG80-2	NC_009328	2	3	1207.5
Geobacillus kaustophilus HTA426	NC_006510	*Geobacillus* WCH70	NC_012793	2	2	238.0
Geobacillus kaustophilus HTA426	NC_006510	*Geobacillus* Y412MC61	NC_013411	1	7	4284.3
Geobacillus kaustophilus HTA426	NC_006510	*Geobacillus* Y412MC10	NC_013406	1	1	465.5
Lactobacillus acidophilus NCFM	NC_006814	*Lactobacillus delbrueckii* ATCC 11842	NC_008054	6	11	2410.8
Lactobacillus acidophilus NCFM	NC_006814	*Lactobacillus helveticus* DPC 4571	NC_010080	3	3	1814.0
Lactobacillus reuteri JCM 1112	NC_010609	*Lactobacillus plantarum* JDM1	NC_012984	2	2	969.5
Leuconostoc citreum KM20	NC_010471	*Leuconostoc mesenteroides* ATCC 8293	NC_008531	1	2	233.0
Listeria innocua Clip11262	NC_003212	*Listeria monocytogenes* Clip81459	NC_012488	1	1	362.5
Staphylococcus aureus RF122	NC_007622	*Staphylococcus epidermidis* ATCC 12228	NC_004461	2	9.5	2333.5
Streptococcus pneumoniae CGSP14	NC_010582	*Streptococcus pneumoniae* JJA	NC_012466	1	3	758.7
Streptococcus pneumoniae CGSP14	NC_010582	*Streptococcus pneumoniae* 19F-14	NC_012469	1	3.5	1326.3
Streptococcus pneumoniae CGSP14	NC_010582	*Streptococcus pneumoniae* D39	NC_008533	1	2.5	822.0
Thermoanaerobacter italicus Ab9	NC_013921	*Thermoanaerobacter tengcongensis* MB4	NC_003869	5	14.5	978.3
Dickeya dadantii Ech586	NC_013592	*Dickeya dadantii* Ech703	NC_012880	3	17.5	4809.8
Dickeya dadantii Ech586	NC_013592	*Dickeya zeae* Ech1591	NC_012912	1	2	336.2
Escherichia coli 536	NC_008253	*Escherichia coli* UMN026	NC_011751	2	10	2905.5
Escherichia coli 536	NC_008253	*Escherichia coli* O111 H 11128	NC_013364	2	73.5	22134.5
Escherichia coli 536	NC_008253	*Escherichia coli* O103 H2 12009	NC_013353	1	4	1243.7
Escherichia coli 536	NC_008253	*Escherichia coli* IAI39	NC_011750	4	313.5	106477.3
Escherichia coli 536	NC_008253	*Escherichia coli* Sakai	NC_002695	2	3	745.8
Escherichia coli 536	NC_008253	*Escherichia coli* APEC O1	NC_008563	1	4.5	2004.5
Escherichia fergusonii ATCC 35469	NC_011740	*Escherichia coli* MG1655	NC_000913	4	37.5	15288.3
Escherichia fergusonii ATCC 35469	NC_011740	*Shigella sonnei* Ss046	NC_007384	4	27.5	8444.3
Edwardsiella ictaluri 93 146	NC_012779	*Edwardsiella tarda* EIB202	NC_013508	3	32	11694.8
Erwinia tasmaniensis Et1/99	NC_010694	*Erwinia pyrifoliae* Ep1 96	NC_012214	2	6	1282.5
Pectobacterium atrosepticum SCRI1043	NC_004547	*Pectobacterium wasabiae* WPP163	NC_013421	3	17.5	7340.2
Photorhabdus luminescens TTO1	NC_005126	*Photorhabdus asymbiotica*	NC_012962	3	17	8162.8
Salmonella enterica Paratyphi AKU 12601	NC_011147	*Salmonella enterica* Paratyphi C RKS4594	NC_012125	3	6.5	1113.8
Salmonella enterica Paratyphi AKU 12601	NC_011147	*Salmonella typhimurium* LT2	NC_003197	3	3	737.3
Shigella sonnei Ss046	NC_007384	*Shigella boydii* Sb227	NC_007613	13	563.5	203959.3
Shigella sonnei Ss046	NC_007384	*Shigella dysenteriae* Sd197	NC_007606	13	523	177708.8
Shigella sonnei Ss046	NC_007384	*Shigella flexneri* 2457T	NC_004741	9	527.5	192208.5
Yersinia pestis Antiqua	NC_008150	*Yersinia pestis* Pestoides F	NC_009381	3	213	71114.0
Yersinia pestis Antiqua	NC_008150	*Yersinia pseudotuberculosis* YPIII	NC_010465	2	87	30288.7
Yersinia pestis Antiqua	NC_008150	*Yersinia pseudotuberculosis* PB1/+	NC_010634	1	83	26410.7
Yersinia pestis Antiqua	NC_008150	*Yersinia enterocolitica* 8081	NC_008800	3	43.5	16635.3
Bacteroides fragilis NCTC 9343	NC_003228	*Bacteroides thetaiotaomicron* VPI-5482	NC_004663	17	43	18197.2
Bradyrhizobium japonicum USDA 110	NC_004463	*Bradyrhizobium* sp. ORS278	NC_009445	10	40.5	13466.7
Geobacter metallireducens GS-15	NC_007517	*Geobacter sulfurreducens* PCA	NC_002939	1	58.5	21379.8
Lactobacillus johnsonii NCC 533	NC_005362	*Lactobacillus acidophilus* NCFM	NC_006814	7	8	2611.3
Leptospira borgpetersenii JB197	NC_008510	*Leptospira borgpetersenii* L550	NC_008508	5	45.5	22827.7
Mycobacterium leprae TN	NC_002677	*Mycobacterium* sp. MCS	NC_008146	2	2	470.0
Nitrobacter hamburgensis X14	NC_007964	*Nitrobacter winogradskyi* Nb-255	NC_007406	13	80.5	26501.2
Rhodopseudomonas palustris BisB18	NC_007925	*Rhodopseudomonas palustris* CGA009	NC_005296	13	110	38206.8
Streptococcus mutans UA159	NC_004350	*Streptococcus agalactiae* 2603V/R	NC_004116	4	6.5	1514.8
Streptomyces avermitilis MA-4680	NC_003155	*Streptomyces coelicolor* A3(2)	NC_003888	4	6	1882.0

Thermus thermophilus HB27	NC_005835	*Thermus thermophilus* HB8	NC_006461	1	3	1320.8
Vibrio cholerae N16961	NC_002505	*Vibrio harveyi* ATCC BAA-1116	NC_009783	3	6.5	1649.8
Mycoplasma agalactiae PG2	NC_009497	*Mycoplasma agalactiae*	NC_013948	1	4.5	1738.8
Mycoplasma hyopneumoniae 232	NC_006360	*Mycoplasma hyopneumoniae* 7448	NC_007332	1	1	303.0
Ureaplasma parvum ATCC 27815	NC_010503	*Ureaplasma urealyticum* ATCC 33699	NC_011374	3	3	1054.0
Thermosipho africanus TCF52B	NC_011653	*Thermosipho melanesiensis* BI429	NC_009616	6	227	69200.3
