# Supplementary material for: Chromosome architecture constrains horizontal gene transfer in bacteria
Source: PLoS Genet. 2018 May 29;14(5):e1007421. doi: 10.1371/journal.pgen.1007421 (PMC5993296; doi:10.1371/journal.pgen.1007421)
Supplement: S3 Table — (PDF) [file pgen.1007421.s004.pdf]

Table S3. Phylogenetic distribution of sources of 17096 insertions

Division	Family	Insertions
**Actinobacteria**		**1595**
	Actinobacteridae	28
	Bifidobacteriaceae	375
	Mycobacteriaceae	1192
**Bacteroidetes**		**625**
	Bacteroidaceae	386
	Porphyromonadaceae	239
**β- Proteobacteria**		**2507**
	Alcaligenaceae	486
	Burkholderiaceae	1468
	Neisseriaceae	543
**Chlamydiae**	Chlamydiaceae	**10**
**∂-Proteobacteria**		**488**
	Desulfovibrionaceae	198
	Myxococcaceae	290
**Firmicutes**		**916**
	Bacillaceae	500
	Unknown	416
**γ-Proteobacteria**		**10831**
	Acidithiobacillaceae	275
	Enterobacteriaceae	5463
	Francisellaceae	468
	Moraxellaceae	753
	Pasteurellaceae	475
	Shewanellaceae	1021
	Vibrionaceae	505
	Xanthomonadaceae	1747
**Spirochaetes**	**Spirochaetaceae**	**124**
