# Supplementary material for: Chromosome architecture constrains horizontal gene transfer in bacteria
Source: PLoS Genet. 2018 May 29;14(5):e1007421. doi: 10.1371/journal.pgen.1007421 (PMC5993296; doi:10.1371/journal.pgen.1007421)
Supplement: S4 Table — (PDF) [file pgen.1007421.s005.pdf]

Table S4. Genomes used to identify 17096 insertions

Division	Family	Organism	Accession	Total Insertion Count	Total Insertion Length
β-Proteobacteria	Acidithiobacillaceae	*Acidithiobacillus ferrooxidans* ATCC 23270	NC_011761	145	342887
γ-Proteobacteria	Acidithiobacillaceae	*Acidithiobacillus ferrooxidans* ATCC 53993	NC_011206	130	291310
γ-Proteobacteria	Moraxellaceae	*Acinetobacter baumannii* AB0057	NC_011586	87	117935
γ-Proteobacteria	Moraxellaceae	*Acinetobacter baumannii* AB307-0294	NC_011595	29	51328
γ-Proteobacteria	Moraxellaceae	*Acinetobacter baumannii* ACICU	NC_010611	95	175337
γ-Proteobacteria	Moraxellaceae	*Acinetobacter baumannii* ATCC 17978	NC_009085	169	392230
γ-Proteobacteria	Moraxellaceae	*Acinetobacter baumannii* AYE	NC_010410	58	116527
γ-Proteobacteria	Moraxellaceae	*Acinetobacter baumannii* SDF	NC_010400	315	559113
γ-Proteobacteria	Pasteurellaceae	*Actinobacillus pleuropneumoniae* L20	NC_009053	71	164667
γ-Proteobacteria	Pasteurellaceae	*Actinobacillus pleuropneumoniae* JL03	NC_010278	55	111393
δ-Proteobacteria	Myxococcaceae	*Anaeromyxobacter dehalogenans* 2CP-1	NC_011891	142	358178
δ-Proteobacteria	Myxococcaceae	*Anaeromyxobacter* sp. K	NC_011145	148	403455
Firmicutes	Bacillaceae	*Bacillus anthracis* CDC 684	NC_012581	11	8968
Firmicutes	Bacillaceae	*Bacillus anthracis* Sterne	NC_005945	5	3652
Firmicutes	Bacillaceae	*Bacillus cereus* 03BB102	NC_012472	42	62735
Firmicutes	Bacillaceae	*Bacillus cereus* AH187	NC_011658	65	118797
Firmicutes	Bacillaceae	*Bacillus cereus* ATCC 10987	NC_003909	144	298347
Firmicutes	Bacillaceae	*Bacillus cereus* E33L	NC_006274	48	115377
Firmicutes	Bacillaceae	*Bacillus cereus* Q1	NC_011969	57	100935
Firmicutes	Bacillaceae	*Bacillus thuringiensis* 97-27	NC_005957	94	157463
Firmicutes	Bacillaceae	*Bacillus thuringiensis* Al Hakam	NC_008600	34	72963
Bacteroidetes	Bacteroidaceae	*Bacteroides fragilis* NCTC 9343	NC_003228	158	479260
Bacteroidetes	Bacteroidaceae	*Bacteroides fragilis* YCH46	NC_006347	228	584608
Actinobacteria	Bifidobacteriaceae	*Bifidobacterium longum*	NC_010816	79	174366
Actinobacteria	Bifidobacteriaceae	*Bifidobacterium longum* NCC2705	NC_004307	56	112166
Actinobacteria	Bifidobacteriaceae	*Bifidobacterium longum* ATCC 15697	NC_011593	240	805305
β-Proteobacteria	Alcaligenaceae	*Bordetella bronchiseptica* RB50	NC_002927	200	613735
β-Proteobacteria	Alcaligenaceae	*Bordetella parapertussis* 12822	NC_002928	48	83620
β Proteobacteria	Alcaligenaceae	*Bordetella pertussis* Tohama I	NC_002929	238	387046
Spirochaetes	Spirochaetaceae	*Borrelia burgdorferi* B31	NC_001318	10	11526
Spirochaetes	Spirochaetaceae	*Borrelia burgdorferi* ZS7	NC_011728	111	844898
Spirochaetes	Spirochaetaceae	*Borrelia garinii* PBi	NC_006156	3	2851
β-Proteobacteria	Burkholderiaceae	*Burkholderia ambifaria* AMMD	NC_008390	95	321255
β-Proteobacteria	Burkholderiaceae	*Burkholderia ambifaria* MC40-6	NC_010551	70	176325
β-Proteobacteria	Burkholderiaceae	*Burkholderia cenocepacia* AU 1054	NC_008060	29	40855
β-Proteobacteria	Burkholderiaceae	*Burkholderia cenocepacia* HI2424	NC_008542	27	71734
β-Proteobacteria	Burkholderiaceae	*Burkholderia cenocepacia* J2315	NC_011000	156	543791

β-Proteobacteria	Burkholderiaceae	*Burkholderia cenocepacia* MC0-3	NC_010508	58	175054
β-Proteobacteria	Burkholderiaceae	*Burkholderia mallei* ATCC 23344	NC_006348	129	224457
β-Proteobacteria	Burkholderiaceae	*Burkholderia mallei* NCTC 10229	NC_008836	101	132701
β-Proteobacteria	Burkholderiaceae	*Burkholderia mallei* NCTC 10247	NC_009080	108	160907
β-Proteobacteria	Burkholderiaceae	*Burkholderia mallei* SAVP1	NC_008785	121	197937
β-Proteobacteria	Burkholderiaceae	*Burkholderia pseudomallei* 1106a	NC_009076	48	95032
β-Proteobacteria	Burkholderiaceae	*Burkholderia pseudomallei* 1710b	NC_007434	270	599274
β-Proteobacteria	Burkholderiaceae	*Burkholderia pseudomallei* 668	NC_009074	79	124434
β-Proteobacteria	Burkholderiaceae	*Burkholderia pseudomallei* K96243	NC_006350	74	241793
β-Proteobacteria	Burkholderiaceae	*Burkholderia thailandensis* E264	NC_007651	103	329917
β-Proteobacteria	Chlamydiaceae	*Chlamydophila pneumoniae* CWL029	NC_000922	6	7510
Chlamydiae	Chlamydiaceae	*Chlamydophila pneumoniae* J138	NC_002491	3	3140
Chlamydiae	Chlamydiaceae	*Chlamydophila pneumoniae* TW-183	NC_005043	1	700
δ-Proteobacteria	Desulfovibrionaceae	*Desulfovibrio vulgaris* DP4	NC_008751	81	213231
δ-Proteobacteria	Desulfovibrionaceae	*Desulfovibrio vulgaris* Hildenborough	NC_002937	117	258299
γ-Proteobacteria	Enterobacteriaceae	*Escherichia coli* 55989	NC_011748	132	257398
γ-Proteobacteria	Enterobacteriaceae	*Escherichia coli* APEC O1	NC_008563	57	104325
γ-Proteobacteria	Enterobacteriaceae	*Escherichia coli* ATCC 8739	NC_010468	59	141512
γ-Proteobacteria	Enterobacteriaceae	*Escherichia coli* CFT073	NC_004431	181	270028
γ-Proteobacteria	Enterobacteriaceae	*Escherichia coli* E24377A	NC_009801	97	207389
γ-Proteobacteria	Enterobacteriaceae	*Escherichia coli* ED1a	NC_011745	193	376153
γ-Proteobacteria	Enterobacteriaceae	*Escherichia coli* HS	NC_009800	75	110903
γ-Proteobacteria	Enterobacteriaceae	*Escherichia coli* LF82	NC_011993	31	81440
γ-Proteobacteria	Enterobacteriaceae	*Escherichia coli* O127:H6 E2348/69	NC_011601	164	368757
γ-Proteobacteria	Enterobacteriaceae	*Escherichia coli* S88	NC_011742	50	90609
γ-Proteobacteria	Enterobacteriaceae	*Escherichia coli* SE11	NC_011415	88	145396
γ-Proteobacteria	Enterobacteriaceae	*Escherichia coli* MG1655	NC_000913	33	36602
γ-Proteobacteria	Enterobacteriaceae	*Escherichia coli* UMN026	NC_011751	170	433281
γ-Proteobacteria	Enterobacteriaceae	*Escherichia coli* UTI89	NC_007946	49	81591
γ-Proteobacteria	Enterobacteriaceae	*Escherichia coli* W3110	AC_000091	49	58753
γ-Proteobacteria	Francisellaceae	*Francisella novicida* U112	NC_008601	80	195913
γ-Proteobacteria	Francisellaceae	*Francisella tularensis*	NC_007880	100	163260
γ-Proteobacteria	Francisellaceae	*Francisella tularensis* FTNF002-00	NC_009749	71	142580
γ-Proteobacteria	Francisellaceae	*Francisella tularensis* OSU18	NC_008369	44	103301
γ-Proteobacteria	Francisellaceae	*Francisella tularensis* FSC147	NC_010677	8	63732
γ-Proteobacteria	Francisellaceae	*Francisella tularensis* FSC198	NC_008245	57	134019
γ-Proteobacteria	Francisellaceae	*Francisella tularensis* SCHU S4	NC_006570	56	129774
γ-Proteobacteria	Francisellaceae	*Francisella tularensis* WY96-3418	NC_009257	52	128818
γ-Proteobacteria	Pasteurellaceae	*Haemophilus influenzae* 86-028NP	NC_007146	42	102480
γ-Proteobacteria	Pasteurellaceae	*Haemophilus influenzae* PittEE	NC_009566	29	46822
γ-Proteobacteria	Pasteurellaceae	*Haemophilus influenzae* PittGG	NC_009567	28	55075

γ-Proteobacteria	Pasteurellaceae	*Haemophilus influenzae* Rd KW20	NC_000907	45	82685
γ-Proteobacteria	Pasteurellaceae	*Haemophilus somnus* 129PT	NC_008309	84	192059
γ-Proteobacteria	Pasteurellaceae	*Haemophilus somnus* 2336	NC_010519	121	417098
γ-Proteobacteria	Enterobacteriaceae	*Klebsiella pneumoniae* 342	NC_011283	277	828349
γ-Proteobacteria	Enterobacteriaceae	*Klebsiella pneumoniae* MGH 78578	NC_009648	166	472740
Actinobacteria	Mycobacteriaceae	*Mycobacterium avium* 104	NC_008595	252	850477
Actinobacteria	Mycobacteriaceae	*Mycobacterium avium* K-10	NC_002944	233	478390
Actinobacteria	Mycobacteriaceae	*Mycobacterium bovis* AF2122/97	NC_002945	31	52708
Actinobacteria	Mycobacteriaceae	*Mycobacterium bovis* BCG 1173P2	NC_008769	32	131937
Actinobacteria	Mycobacteriaceae	*Mycobacterium bovis* BCG 172	NC_012207	26	81278
Actinobacteria	Mycobacteriaceae	*Mycobacterium* sp. JLS	NC_009077	135	612930
Actinobacteria	Mycobacteriaceae	*Mycobacterium* sp. KMS	NC_008705	45	87732
Actinobacteria	Mycobacteriaceae	*Mycobacterium* sp. MCS	NC_008146	46	64932
Actinobacteria	Mycobacteriaceae	*Mycobacterium tuberculosis* CDC1551	NC_002755	67	71725
Actinobacteria	Mycobacteriaceae	*Mycobacterium tuberculosis* F11	NC_009565	43	53037
Actinobacteria	Mycobacteriaceae	*Mycobacterium tuberculosis* H37Ra	NC_009525	36	41478
Actinobacteria	Mycobacteriaceae	*Mycobacterium tuberculosis* H37Rv	NC_000962	43	49646
Actinobacteria	Mycobacteriaceae	*Mycobacterium ulcerans* Agy99	NC_008611	203	592617
β-Proteobacteria	Neisseriaceae	*Neisseria gonorrhoeae* FA 1090	NC_002946	82	90360
β-Proteobacteria	Neisseriaceae	*Neisseria gonorrhoeae* NCCP11945	NC_011035	197	274954
β-Proteobacteria	Neisseriaceae	*Neisseria meningitidis* 053442	NC_010120	60	92546
β-Proteobacteria	Neisseriaceae	*Neisseria meningitidis* FAM18	NC_008767	69	133332
β-Proteobacteria	Neisseriaceae	*Neisseria meningitidis* MC58	NC_003112	83	168070
β-Proteobacteria	Neisseriaceae	*Neisseria meningitidis* Z2491	NC_003116	52	75857
Bacteroidetes	Porphyromonadaceae	*Porphyromonas gingivalis* ATCC 33277	NC_010729	135	330993
Bacteroidetes	Porphyromonadaceae	*Porphyromonas gingivalis* W83	NC_002950	104	262422
γ-Proteobacteria	Enterobacteriaceae	*Salmonella enterica* Agona SL483	NC_011149	54	97122
γ-Proteobacteria	Enterobacteriaceae	*Salmonella enterica* Choleraesuis SC-B67	NC_006905	44	72938
γ-Proteobacteria	Enterobacteriaceae	*Salmonella enterica* CT_02021853	NC_011205	14	25285
γ-Proteobacteria	Enterobacteriaceae	*Salmonella enterica* Enteritidis P125109	NC_011294	16	25346
γ-Proteobacteria	Enterobacteriaceae	*Salmonella enterica* Gallinarum 287/91	NC_011274	11	26030
γ-Proteobacteria	Enterobacteriaceae	*Salmonella enterica* Heidelberg SL476	NC_011083	53	112442
γ-Proteobacteria	Enterobacteriaceae	*Salmonella enterica* Newport SL254	NC_011080	31	52228
γ-Proteobacteria	Enterobacteriaceae	*Salmonella enterica* Paratyphi A AKU_12601	NC_011147	22	33072
γ-Proteobacteria	Enterobacteriaceae	*Salmonella enterica* Paratyphi B SPB7	NC_010102	58	90319
γ-Proteobacteria	Enterobacteriaceae	*Salmonella enterica* Paratyphi C RKS4594	NC_012125	32	54709
γ-Proteobacteria	Enterobacteriaceae	*Salmonella enterica* Schwarzengrund CVM19633	NC_011094	52	106414
γ-Proteobacteria	Enterobacteriaceae	*Salmonella enterica* Typhi Ty2	NC_004631	71	92397
γ-Proteobacteria	Enterobacteriaceae	*Salmonella enterica* Typhimurium LT2	NC_003197	34	73966
γ-Proteobacteria	Shewanellaceae	*Shewanella baltica* OS155	NC_009052	202	511463
γ-Proteobacteria	Shewanellaceae	*Shewanella baltica* OS185	NC_009665	110	230130

γ-Proteobacteria	Shewanellaceae	*Shewanella baltica* OS195	NC_009997	136	294359
γ-Proteobacteria	Shewanellaceae	*Shewanella baltica* OS223	NC_011663	128	341891
γ-Proteobacteria	Shewanellaceae	*Shewanella putrefaciens* CN-32	NC_009438	108	364829
γ-Proteobacteria	Shewanellaceae	*Shewanella* sp. MR-4	NC_008321	94	252684
γ-Proteobacteria	Shewanellaceae	*Shewanella* sp. MR-7	NC_008322	105	317538
γ-Proteobacteria	Shewanellaceae	*Shewanella* sp. W3-18-1	NC_008750	138	410048
γ-Proteobacteria	Enterobacteriaceae	*Shigella boydii* CDC 3083-94	NC_010658	308	437683
γ-Proteobacteria	Enterobacteriaceae	*Shigella boydii* Sb227	NC_007613	356	467700
γ-Proteobacteria	Enterobacteriaceae	*Shigella dysenteriae* Sd197	NC_007606	204	289662
γ-Proteobacteria	Enterobacteriaceae	*Shigella flexneri* 2a 2457T	NC_004741	246	271136
γ-Proteobacteria	Enterobacteriaceae	*Shigella flexneri* 2a 301	NC_004337	197	251486
γ-Proteobacteria	Enterobacteriaceae	*Shigella flexneri* 5 8401	NC_008258	256	302643
γ-Proteobacteria	Enterobacteriaceae	*Shigella sonnei* Ss046	NC_007384	352	677856
Firmicutes	Unknown	*Staphylococcus aureus* RF122	NC_007622	33	63044
Firmicutes	Unknown	*Staphylococcus aureus* COL	NC_002951	7	15746
Firmicutes	Unknown	*Staphylococcus aureus* JH1	NC_009632	19	34903
Firmicutes	Unknown	*Staphylococcus aureus* JH9	NC_009487	19	34307
Firmicutes	Unknown	*Staphylococcus aureus* MRSA252	NC_002952	52	102649
Firmicutes	Unknown	*Staphylococcus aureus* MSSA476	NC_002953	5	9978
Firmicutes	Unknown	*Staphylococcus aureus* Mu3	NC_009782	21	30887
Firmicutes	Unknown	*Staphylococcus aureus* Mu50	NC_002758	22	31736
Firmicutes	Unknown	*Staphylococcus aureus* MW2	NC_003923	3	3124
Firmicutes	Unknown	*Staphylococcus aureus* N315	NC_002745	23	49671
Firmicutes	Unknown	*Staphylococcus aureus* NCTC 8325	NC_007795	13	14765
Firmicutes	Unknown	*Staphylococcus aureus* Newman	NC_009641	22	55272
Firmicutes	Unknown	*Staphylococcus aureus* USA300	NC_010079	2	2204
Firmicutes	Unknown	*Staphylococcus epidermidis* ATCC 12228	NC_004461	88	169801
Firmicutes	Unknown	*Staphylococcus epidermidis* RP62A	NC_002976	87	221827
Actinobacteria	Actinobacteridae	*Tropheryma whipplei* Twist	NC_004572	12	20540
Actinobacteria	Actinobacteridae	*Tropheryma whipplei* TW08/27	NC_004551	16	27040
γ-Proteobacteria	Vibrionaceae	*Vibrio cholerae* M66-2	NC_012578	11	13547
γ-Proteobacteria	Vibrionaceae	*Vibrio cholerae* O1 N16961	NC_002505	30	61076
γ-Proteobacteria	Vibrionaceae	*Vibrio cholerae* O395	NC_009457	67	131864
γ-Proteobacteria	Vibrionaceae	*Vibrio fischeri* ES114	NC_006840	50	138694
γ-Proteobacteria	Vibrionaceae	*Vibrio fischeri* MJ11	NC_011184	56	157066
γ-Proteobacteria	Vibrionaceae	*Vibrio vulnificus* CMCP6	NC_004459	142	301123
γ-Proteobacteria	Vibrionaceae	*Vibrio vulnificus* YJ016	NC_005139	149	358774
γ-Proteobacteria	Xanthomonadaceae	*Xanthomonas axonopodis* 306	NC_003919	230	605331
γ-Proteobacteria	Xanthomonadaceae	*Xanthomonas campestris* 85-10	NC_007508	282	617433
γ-Proteobacteria	Xanthomonadaceae	*Xanthomonas oryzae* KACC10331	NC_006834	267	443767
γ-Proteobacteria	Xanthomonadaceae	*Xanthomonas oryzae* MAFF 311018	NC_007705	348	531633

γ-Proteobacteria	Xanthomonadaceae	*Xanthomonas oryzae* PXO99A	NC_010717	358	922954
γ-Proteobacteria	Xanthomonadaceae	*Xylella fastidiosa* 9a5c	NC_002488	132	253159
γ-Proteobacteria	Xanthomonadaceae	*Xylella fastidiosa* M12	NC_010513	57	109043
γ-Proteobacteria	Xanthomonadaceae	*Xylella fastidiosa* M23	NC_010577	41	69314
γ-Proteobacteria	Xanthomonadaceae	*Xylella fastidiosa* Temecula1	NC_004556	32	53447
γ-Proteobacteria	Enterobacteriaceae	*Yersinia pestis* Angola	NC_010159	172	251086
γ-Proteobacteria	Enterobacteriaceae	*Yersinia pestis* Antiqua	NC_008150	172	247598
γ-Proteobacteria	Enterobacteriaceae	*Yersinia pestis* 91001	NC_005810	104	162239
γ-Proteobacteria	Enterobacteriaceae	*Yersinia pestis* CO92	NC_003143	131	213313
γ-Proteobacteria	Enterobacteriaceae	*Yersinia pestis* KIM	NC_004088	119	181220
γ-Proteobacteria	Enterobacteriaceae	*Yersinia pestis* Nepal516	NC_008149	128	160739
γ-Proteobacteria	Enterobacteriaceae	*Yersinia pestis* Pestoides F	NC_009381	107	129681
γ-Proteobacteria	Enterobacteriaceae	*Yersinia pseudotuberculosis* IP 31758	NC_009708	70	171600
γ-Proteobacteria	Enterobacteriaceae	*Yersinia pseudotuberculosis* IP 32953	NC_006155	45	96718
γ-Proteobacteria	Enterobacteriaceae	*Yersinia pseudotuberculosis* PB1/+	NC_010634	39	70161
γ-Proteobacteria	Enterobacteriaceae	*Yersinia pseudotuberculosis* YPIII	NC_010465	94	149002
