# Supplementary material for: Chromosome architecture constrains horizontal gene transfer in bacteria
Source: PLoS Genet. 2018 May 29;14(5):e1007421. doi: 10.1371/journal.pgen.1007421 (PMC5993296; doi:10.1371/journal.pgen.1007421)
Supplement: S5 Table — (PDF) [file pgen.1007421.s006.pdf]

Table S5. Predicted replication breakpoints

Organism	Accession	Length	Origin[1]	Terminus[1]	Dif	Delta
Acidithiobacillus ferrooxidans ATCC 23270	NC_011761	2982397	2236	1704353	1713165	-0.295
Acinetobacter baumannii AB307-0294	NC_011595	3760981	3759848	1849034	1842270	0.180
Actinobacillus pleuropneumoniae L20	NC_009053	2274482	1886036	742280	742280	0.000
Anaeromyxobacter dehalogenans 2CP-1	NC_011891	5029329	247	2538375	2536100	0.045
Aster yellows witches'-broom AYWB	NC_007716	706569	706569	260156		
Bacteroides fragilis NCTC 9343	NC_003228	5205140	5203757	2729356	2795490	-1.271
Bifidobacterium longum	NC_010816	2375792	1668586	561017	560930	0.004
Borrelia burgdorferi B31	NC_001318	910724	458276	1	1	0.000
Brucella abortus S19	NC_010742	2122487	2009256	1014214	1049890	-1.681
Buchnera aphidicola str. Tuc7	NC_011834	641895	627408	342257		
Burkholderia pseudomallei 1710b	NC_007434	4126292	230307	2390881	2390580	0.007
Campylobacter jejuni subsp. jejuni 81-176	NC_008787	1616554	1482	814822		
Clostridium botulinum A str. Hall	NC_009698	3760560	3760560	1839389		
Clostridium perfringens ATCC 13124	NC_008261	3256683	3255309	1540393		
Coxiella burnetii RSA 331	NC_010117	2016427	1935567	920401		
Desulfovibrio vulgaris str. Hildenborough	NC_002937	3570858	1754275	1	5261	-0.147
Ehrlichia ruminantium str. Welgevonden	NC_005295	1516355	19809	718954	747980	-1.914
Escherichia coli CFT073	NC_004431	5231428	4427044	1781770	1781770	0.000
Escherichia coli str. K12 substr. MG1655	NC_000913	4639675	3924034	1588790	1588790	0.000
Francisella novicida U112	NC_008601	1910031	1908196	926219	918785	0.389
Haemophilus somnus 129PT	NC_008309	2007700	1935344	953380	953380	0.000
Helicobacter pylori HPAG1	NC_008086	1596366	1438896	653439		
Klebsiella pneumoniae sMGH 78578	NC_009648	5315120	4542931	1892080	1892080	0.000
Lactococcus lactis subsp. cremoris MG1363	NC_009004	2529478	2529478	1232525		
Legionella pneumophila sstr. Philadelphia 1	NC_002942	3397754	3395274	1734249		
Leptospira borgpetersenii Hardjo-bovis JB197	NC_008510	3576473	3576473	1661700		
Mycobacterium sp. MCS	NC_008146	5705448	2068	2760365	2760365	0.000
Mycobacterium tuberculosis H37Rv	NC_000962	4411532	4411532	2232655	2232655	0.000
Neisseria meningitidis FAM18	NC_008767	2194961	1260	1097552	1101360	-0.173
Porphyromonas gingivalis ATCC 33277	NC_010729	2354886	2242943	748876	284445	19.722
Rhodobacter sphaeroides 2.4.1	NC_007493	3188609	3029036	1434944	1436845	-0.060
Rhodopseudomonas palustris CGA009	NC_005296	5459213	337337	3153460	3156850	-0.062
Rickettsia prowazekii str. Madrid E	NC_000963	1111523	1109104	621153	596110	2.253
Salmonella typhimurium LT2	NC_003197	4857432	4084165	1629690	1629690	0.000
Shigella flexneri 2a str. 2457T	NC_004741	4599354	3965820	1651225	1651225	0.000
Staphylococcus aureus subsp. aureus COL	NC_002951	2809422	2808623	1423950	1423950	0.000
Streptococcus agalactiae 2603V/R	NC_004116	2160267	2151551	987102		
Streptococcus equi subsp. zooepidemicus	NC_012470	2149868	2148860	1080727		
Streptococcus pneumoniae CGSP14	NC_010582	2209198	2207172	1108214		
Streptococcus pyogenes MGAS10394	NC_006086	1899877	1888741	899849		
Streptococcus suis 05ZYH33	NC_009442	2096309	2095341	854770		
Streptococcus thermophilus CNRZ1066	NC_006449	1796226	1795404	905801		
Thermoanaerobacter pseudethanolicus ATCC33223	NC_010321	2362816	78	1228591		
Treponema pallidum subsp. pallidum SS14	NC_010741	1139457	3	557758		
Tropheryma whipplei TW08/27	NC_004551	925938	2889	484011	478960	0.546
Vibrio cholerae O1 biovar eltor str. N16961	NC_002505	2961149	371	1564115	1564115	0.000
Vibrio fischeri ES114	NC_006840	2897536	312	1446515	1446515	0.000
Vibrio vulnificus YJ016	NC_005139	3354505	3352490	1722010	1722010	0.000
Wolbachia sp. wRi	NC_012416	1445873	20789	564524	285010	19.332
Xanthomonas campestris str. 85-10	NC_007508	5178466	5061009	2782425	2782425	0.000
Xylella fastidiosa M23	NC_010577	2535690	2534894	1141721	1143850	-0.084
Yersinia pseudotuberculosis YPIII	NC_010465	4689441	4655532	2187970	2187970	0.000
Acidithiobacillus ferrooxidans ATCC 53993	NC_011206	2885038	4041	1585274	1594270	-0.312
Acinetobacter baumannii AB0057	NC_011586	4050513	21686	2150359	2150170	0.005
Acinetobacter baumannii ACICU	NC_010611	3904116	5369	1929027	1936435	-0.190
Acinetobacter baumannii ATCC 17978	NC_009085	3976747	295	2034289	2033610	0.017
Acinetobacter baumannii AYE	NC_010410	3936291	3935233	1897266	1898710	-0.037
Acinetobacter baumannii SDF	NC_010400	3421954	3421092	1853510	1839405	0.412
Actinobacillus pleuropneumoniae str. JL03	NC_010278	2242062	1849019	695450	695450	0.000
Anaeromyxobacter sp. K	NC_011145	5061632	4965750	2472569	2468350	0.083
Bacillus anthracis str. CDC 684	NC_012581	5230115	280	1731230	1731230	0.000
Bacillus anthracis str. Sterne	NC_005945	5228663	407	2507809	2507809	0.000
Bacillus cereus 03BB102	NC_012472	5269628	291	2592760	2592760	0.000
Bacillus cereus AH187	NC_011658	5269030	408	2564400	2564400	0.000
Bacillus cereus ATCC 10987	NC_003909	5224283	407	2590340	2590340	0.000

Bacillus cereus E33L	NC_006274	5300915	407	2571015	2571015	0.000
Bacillus cereus Q1	NC_011969	5214195	407	2510630	2510630	0.000
Bacillus thuringiensis serovar konkukian str. 97-27	NC_005957	5237682	408	2560270	2560270	0.000
Bacillus thuringiensis str. Al Hakam	NC_008600	5257091	350	2593005	2593005	0.000
Bacteroides fragilis YCH46	NC_006347	5277274	5275891	2614583	2680710	-1.253
Bifidobacterium longum NCC2705	NC_004307	2256640	1598655	532426	529450	0.132
Bifidobacterium longum ATCC 15697	NC_011593	2832748	2831798	1384739	1519770	-4.767
Bordetella bronchiseptica RB50	NC_002927	5339179	5330937	2978346	2955855	0.421
Bordetella parapertussis 12822	NC_002928	4773551	4765261	2891411	2905100	-0.287
Bordetella pertussis Tohama I	NC_002929	4086189	3645063	2226900	2229070	-0.053
Borrelia burgdorferi ZS7	NC_011728	906707	454374	1	1	0.000
Borrelia garinii PBi	NC_006156	904246	460483	1	1	0.000
Brucella abortus biovar 1 str. 9-941	NC_006932	2124241	2008959	1015915	1051560	-1.678
Brucella canis ATCC 23365	NC_010103	2105969	1990465	995373	1032845	-1.779
Brucella melitensis 16M	NC_003317	2117144	1285	990888	954740	1.707
Brucella melitensis ATCC 23457	NC_012441	2125701	2012210	1025600	1053120	-1.295
Brucella melitensis biovar Abortus 2308	NC_007618	2121359	2008089	1013035	1048710	-1.682
Brucella ovis ATCC 25840	NC_009505	2111370	1997935	1015890	1043450	-1.305
Brucella suis 1330	NC_004310	2107794	1992297	1006805	1034390	-1.309
Brucella suis ATCC 23445	NC_010169	1923763	1810101	1016674	1053980	-1.939
Buchnera aphidicola str. APS	NC_002528	640681	633979	340820		
Burkholderia ambifaria AMMD	NC_008390	3556545	66180	1806427	1814910	-0.239
Burkholderia ambifaria MC40-6	NC_010551	3443583	74162	1809724	1837540	-0.808
Burkholderia cenocepacia AU 1054	NC_008060	3294563	2665	1334396	1347910	-0.410
Burkholderia cenocepacia HI2424	NC_008542	3483902	67229	1877716	1891230	-0.388
Burkholderia cenocepacia J2315	NC_011000	3870082	430	1899347	1957390	-1.500
Burkholderia cenocepacia MC0-3	NC_010508	3532883	102201	1853428	1868540	-0.428
Burkholderia mallei ATCC 23344	NC_006348	3510148	2993264	1079374	1081310	-0.055
Burkholderia mallei NCTC 10229	NC_008836	3458208	2265868	156590	157670	-0.031
Burkholderia mallei NCTC 10247	NC_009080	3495687	2195663	995332	999300	-0.114
Burkholderia mallei SAVP1	NC_008785	3497479	479342	1460785	1455290	0.157
Burkholderia pseudomallei 1106a	NC_009076	3988455	1160	1990079	2013530	-0.588
Burkholderia pseudomallei 668	NC_009074	3912947	1160	1984624	2008010	-0.598
Burkholderia pseudomallei K96243	NC_006350	4074542	4074542	2037624	1993990	1.071
Burkholderia thailandensis E264	NC_007651	3809201	1160	2650432	2649010	0.037
Campylobacter jejuni RM1221	NC_003912	1777831	1482	894711		
Campylobacter jejuni subsp. jejuni 81116	NC_009839	1628115	1482	820226		
Campylobacter jejuni subsp. jejuni NCTC 11168	NC_002163	1641481	1482	814350		
Chlamydophila pneumoniae CWL029	NC_000922	1230230	841974	208413	208370	0.003
Chlamydophila pneumoniae J138	NC_002491	1226565	841295	207596	207990	-0.032
Chlamydophila pneumoniae TW-183	NC_005043	1225935	839280	205380	205775	-0.032
Clostridium botulinum A2 str. Kyoto	NC_012563	4155278	4154149	2008417		
Clostridium botulinum A3 str. Loch Maree	NC_010520	3992906	13	1969683		
Clostridium botulinum B1 str. Okra	NC_010516	3958233	3956985	1909159		
Clostridium botulinum F str. Langeland	NC_009699	3995387	3994102	1914642		
Clostridium perfringens str. 13	NC_003366	3031430	3030055	1358666		
Coxiella burnetii CbuG_Q212	NC_011527	2008870	295087	1056841		
Coxiella burnetii CbuK_Q154	NC_011528	2063100	203054	866000		
Coxiella burnetii Dugway 7E9-12	NC_009727	2158758	179691	1140893		
Coxiella burnetii RSA 493	NC_002971	1995281	36619	866869		
Desulfovibrio vulgaris DP4	NC_008751	3462887	1765267	3442	3443	0.000
Ehrlichia ruminantium str. Gardel	NC_006831	1499920	956	694430	723500	-1.938
Escherichia coli 55989	NC_011748	5154862	4306304	1706400	1706400	0.000
Escherichia coli APEC O1	NC_008563	5082025	4219676	1643810	1643810	0.000
Escherichia coli ATCC 8739	NC_010468	4746218	4704354	2353090	2353090	0.000
Escherichia coli E24377A	NC_009801	4979619	4247908	1701170	1701170	0.000
Escherichia coli ED1a	NC_011745	5209548	4362816	1635440	1635440	0.000
Escherichia coli HS	NC_009800	4643538	3953190	1607585	1607585	0.000
Escherichia coli LF82	NC_011993	4773108	3972000	1552240	1552240	0.000
Escherichia coli O127:H6 str. E2348/69	NC_011601	4965553	4222630	1702590	1702590	0.000
Escherichia coli S88	NC_011742	5032268	4134384	1591015	1591015	0.000
Escherichia coli SE11	NC_011415	4887515	4180160	1691120	1691120	0.000
Escherichia coli UMN026	NC_011751	5202090	4412878	1799810	1799810	0.000
Escherichia coli UTI89	NC_007946	5065741	4186879	1653005	1653005	0.000
Escherichia coli W3110	AC_000091	4646332	3711047	1592475	1592475	0.000
Francisella tularensis subsp. holarctica	NC_007880	1895994	1894286	1192572	1166760	1.361
Francisella tularensis FTNF002-00	NC_009749	1890909	1888927	1192480	1167160	1.339
Francisella tularensis subsp. holarctica OSU18	NC_008369	1895727	1893745	1173313	1170715	0.137
Francisella tularensis subsp. mediasiatica FSC147	NC_010677	1893886	1891955	1052132	1049140	0.158
Francisella tularensis subsp. tularensis FSC198	NC_008245	1892616	1831690	1001966	994640	0.387

Francisella tularensis subsp. tularensis SCHU S4	NC_006570	1892775	1831849	1002013	994690	0.387
Francisella tularensis subsp. tularensis WY96-3418	NC_009257	1898476	1804508	863624	856205	0.391
Haemophilus influenzae 86-028NP	NC_007146	1914490	792372	1623110	1623110	0.000
Haemophilus influenzae PittEE	NC_009566	1813033	6963	932945	932945	0.000
Haemophilus influenzae PittGG	NC_009567	1887192	1256069	123620	123620	0.000
Haemophilus influenzae Rd KW20	NC_000907	1830138	612623	1473975	1473975	0.000
Haemophilus somnus 2336	NC_010519	2263857	2153804	1535030	1535030	0.000
Helicobacter pylori 26695	NC_000915	1667867	1636479	715088		
Helicobacter pylori G27	NC_011333	1652982	1546161	781545		
Helicobacter pylori J99	NC_000921	1643831	1543495	678871		
Helicobacter pylori P12	NC_011498	1673813	1564811	820634		
Helicobacter pylori Shi470	NC_010698	1608548	1525581	579250		
Klebsiella pneumoniae 342	NC_011283	5641239	5603011	2724485	2724485	0.000
Lactococcus lactis subsp. cremoris SK11	NC_008527	2438589	143	1211333		
Legionella pneumophila str. Corby	NC_009494	3576470	3575882	1067437		
Legionella pneumophila str. Lens	NC_006369	3345687	3344649	1623139		
Legionella pneumophila str. Paris	NC_006368	3503610	3502582	1700183		
Leptospira borgpetersenii serovar Hardjo-bovis L550	NC_008508	3614446	3557014	1902513		
Leptospira interrogans str. Fiocruz L1-130	NC_005823	4277185	291	2132879		
Leptospira interrogans serovar Lai str. 56601	NC_004342	4332241	233	2149582		
Mycobacterium avium 104	NC_008595	5475491	33418	2723920	2723920	0.000
Mycobacterium avium subsp. paratuberculosis K-10	NC_002944	4829781	3929270	1888590	1888590	0.000
Mycobacterium bovis AF2122/97	NC_002945	4345492	4345492	2211570	2211570	0.000
Mycobacterium bovis BCG str. Pasteur 1173P2	NC_008769	4374522	29667	2211310	2211310	0.000
Mycobacterium bovis BCG str. Tokyo 172	NC_012207	4371711	4371711	2204101	2204101	0.000
Mycobacterium marinum M	NC_010612	6636827	6636827	3309292		
Mycobacterium sp. JLS	NC_009077	6048425	34182	2753145	2753145	0.000
Mycobacterium sp. KMS	NC_008705	5737227	7983	2778255	2778255	0.000
Mycobacterium tuberculosis CDC1551	NC_002755	4403837	4403837	2230001	2230001	0.000
Mycobacterium tuberculosis F11	NC_009565	4424435	101	2240905	2240905	0.000
Mycobacterium tuberculosis H37Ra	NC_009525	4419977	4419977	2242555	2242555	0.000
Mycobacterium ulcerans Agy99	NC_008611	5631606	412513	2481900	2481900	0.000
Mycoplasma hyopneumoniae 232	NC_006360	892758	224079	457333		
Mycoplasma hyopneumoniae 7448	NC_007332	920079	219534	467401		
Mycoplasma hyopneumoniae J	NC_007295	897405	213711	450863		
Neisseria gonorrhoeae FA 1090	NC_002946	2153922	1891591	778407	778170	0.011
Neisseria gonorrhoeae NCCP11945	NC_011035	2232025	1942202	864562	835985	1.280
Neisseria meningitidis 053442	NC_010120	2153416	15711	1094193	1090930	0.152
Neisseria meningitidis MC58	NC_003112	2272360	12173	1229188	1229350	-0.007
Neisseria meningitidis Z2491	NC_003116	2184406	245991	1290249	1290410	-0.007
Onion yellows phytoplasma OY-M	NC_005303	853092	853092	492102		
Porphyromonas gingivalis W83	NC_002950	2343476	1	1185142	1544496	-15.334
Prochlorococcus marinus str. MIT 9303	NC_008820	2682675	1667499	2681411		
Prochlorococcus marinus str. MIT 9313	NC_005071	2410873	551717	2409527		
Rhodobacter sphaeroides ATCC 17029	NC_009049	3147721	3097265	1482864	1483625	-0.024
Rhodobacter sphaeroides KD131	NC_011963	3152792	2702674	1105904	1102435	0.110
Rhodopseudomonas palustris TIE-1	NC_011004	5744041	318977	3361142	3368055	-0.120
Rickettsia bellii OSU 85-389	NC_009883	1528980	6019	769410	772690	-0.215
Rickettsia bellii RML369-C	NC_007940	1522076	848	763697	755670	0.527
Rickettsia conorii str. Malish 7	NC_003103	1268755	1263557	642388	696670	-4.278
Rickettsia felis URRWXCal2	NC_007109	1485148	11600	881279	887520	-0.420
Rickettsia massiliae MTU5	NC_009900	1360898	1293387	809695	810920	-0.090
Rickettsia typhi str. Wilmington	NC_006142	1111496	139125	563953	599255	-3.176
Salmonella enterica Agona str. SL483	NC_011149	4798660	4001832	1585920	1585920	0.000
Salmonella enterica Choleraesuis str. SC-B67	NC_006905	4755700	4019220	1652790	1652790	0.000
Salmonella enterica Dublin str. CT_02021853	NC_011205	4842908	4112721	1737140	1737140	0.000
Salmonella enterica Enteritidis str. P125109	NC_011294	4685848	3954503	1602415	1602415	0.000
Salmonella enterica Gallinarum str. 287/91	NC_011274	4658697	3744848	1651270	1651270	0.000
Salmonella enterica Heidelberg str. SL476	NC_011083	4888768	4094056	1676820	1676820	0.000
Salmonella enterica Newport str. SL254	NC_011080	4827641	4050430	1626660	1626660	0.000
Salmonella enterica Paratyphi A str. AKU_12601	NC_011147	4581797	3854863	1395400	1395400	0.000
Salmonella enterica Paratyphi B str. SPB7	NC_010102	4858887	4028738	1493990	1493990	0.000
Salmonella enterica Paratyphi C strain RKS4594	NC_012125	4833080	4015964	2247170	2247170	0.000
Salmonella enterica Schwarzengrund str. CVM19633	NC_011094	4709075	3960176	1597350	1597350	0.000
Salmonella enterica Typhi str. Ty2	NC_004631	4791961	3750926	1513310	1513310	0.000
Shewanella baltica OS155	NC_009052	5127376	5122413	2542700	2542700	0.000
Shewanella baltica OS185	NC_009665	5229686	5225065	2624900	2624900	0.000
Shewanella baltica OS195	NC_009997	5347283	5342662	2711560	2711560	0.000
Shewanella baltica OS223	NC_011663	5145902	5140976	2625285	2625285	0.000
Shewanella putrefaciens CN-32	NC_009438	4659220	4653761	2284430	2284430	0.000

Shewanella sp. MR-4	NC_008321	4706287	4699817	2387369	2387369	0.000
Shewanella sp. MR-7	NC_008322	4792610	4786141	2327486	2327486	0.000
Shewanella sp. W3-18-1	NC_008750	4708380	4670960	2328996	2328996	0.000
Shigella boydii CDC 3083-94	NC_010658	4615997	3904670	1608805	1608805	0.000
Shigella boydii Sb227	NC_007613	4519823	3665644	1539620	1539620	0.000
Shigella dysenteriae Sd197	NC_007606	4369232	4288449	1482380	1482380	0.000
Shigella flexneri 2a str. 301	NC_004337	4607203	3809950	1611585	1611585	0.000
Shigella flexneri 5 str. 8401	NC_008258	4574284	3858677	1603280	1603280	0.000
Shigella sonnei Ss046	NC_007384	4825265	3925631	1698240	1698240	0.000
Staphylococcus aureus RF122	NC_007622	2742531	2741705	1348530	1348530	0.000
Staphylococcus aureus subsp. aureus JH1	NC_009632	2906507	2905805	1507345	1507345	0.000
Staphylococcus aureus subsp. aureus JH9	NC_009487	2906700	2905929	1507470	1507470	0.000
Staphylococcus aureus subsp. aureus MRSA252	NC_002952	2902619	516	1447630	1447630	0.000
Staphylococcus aureus subsp. aureus MSSA476	NC_002953	2799802	2798976	1413430	1413430	0.000
Staphylococcus aureus subsp. aureus Mu3	NC_009782	2880168	2879342	1460620	1460620	0.000
Staphylococcus aureus subsp. aureus Mu50	NC_002758	2878529	2877703	1459220	1459220	0.000
Staphylococcus aureus subsp. aureus MW2	NC_003923	2820462	2819636	1384891	1384891	0.000
Staphylococcus aureus subsp. aureus N315	NC_002745	2814816	2813990	1382903	1382903	0.000
Staphylococcus aureus subsp. aureus NCTC 8325	NC_007795	2821361	2820535	1320180	1320180	0.000
Staphylococcus aureus subsp. aureus str. Newman	NC_009641	2878897	2878071	1420130	1420130	0.000
Staphylococcus aureus subsp. aureus USA300	NC_010079	2872915	2872116	1414100	1414100	0.000
Staphylococcus epidermidis ATCC 12228	NC_004461	2499279	2498258	1070730	1070730	0.000
Staphylococcus epidermidis RP62A	NC_002976	2616530	835	964800	964800	0.000
Streptococcus agalactiae A909	NC_007432	2127839	2119224	1045288		
Streptococcus agalactiae NEM316	NC_004368	2211485	2202843	1009880		
Streptococcus equi subsp. equi 4047	NC_012471	2253793	2244383	1169155		
Streptococcus equi MGCS10565	NC_011134	2024171	2023396	1050156		
Streptococcus pneumoniae ATCC 700669	NC_011900	2221315	2219290	1039514		
Streptococcus pneumoniae D39	NC_008533	2046115	2045144	1047608		
Streptococcus pneumoniae G54	NC_011072	2078953	2078178	1035465		
Streptococcus pneumoniae Hungary19A-6	NC_010380	2245615	2244840	1186653		
Streptococcus pneumoniae JJA	NC_012466	2120234	2118220	1040158		
Streptococcus pneumoniae P1031	NC_012467	2111882	2111107	1120988		
Streptococcus pneumoniae Taiwan19F-14	NC_012469	2112148	22190	1132060		
Streptococcus pneumoniae TIGR4	NC_003028	2160842	2158828	1094108		
Streptococcus pyogenes M1 GAS	NC_002737	1852441	231	978408		
Streptococcus pyogenes MGAS10270	NC_008022	1928252	201	971621		
Streptococcus pyogenes MGAS10750	NC_008024	1937111	201	1002798		
Streptococcus pyogenes MGAS2096	NC_008023	1860355	1850274	928145		
Streptococcus pyogenes MGAS315	NC_004070	1900521	1890277	891347		
Streptococcus pyogenes MGAS5005	NC_007297	1838554	201	896801		
Streptococcus pyogenes MGAS6180	NC_007296	1897573	1887493	897704		
Streptococcus pyogenes MGAS8232	NC_003485	1895017	201	954308		
Streptococcus pyogenes MGAS9429	NC_008021	1836467	1826398	966924		
Streptococcus pyogenes NZ131	NC_011375	1815785	1805527	931223		
Streptococcus pyogenes SSI-1	NC_004606	1894275	1884982	1021867		
Streptococcus pyogenes str. Manfredo	NC_009332	1841271	201	896367		
Streptococcus suis 98HAH33	NC_009443	2095698	2095698	855632		
Streptococcus thermophilus LMD-9	NC_008532	1856368	1847017	935409		
Streptococcus thermophilus LMG 18311	NC_006448	1796846	1796024	903678		
Thermoanaerobacter sp. X514	NC_010320	2457259	194	1635064		
Treponema pallidum subsp. pallidum str. Nichols	NC_000919	1138011	3	556326		
Tropheryma whipplei str. Twist	NC_004572	927303	2889	409388	414350	-0.535
Vibrio cholerae M66-2	NC_012578	2892523	371	1521560	1521560	0.000
Vibrio cholerae O395	NC_009457	3024069	2669429	1114650	1114650	0.000
Vibrio fischeri MJ11	NC_011184	2905029	312	1461515	1461515	0.000
Vibrio vulnificus CMCP6	NC_004459	3281944	1011899	2661300	2661300	0.000
Wolbachia endosymbiont of D. melanogaster	NC_002978	1267782	1066067	344757	438690	-7.409
Xanthomonas axonopodis pv. citri str. 306	NC_003919	5175554	5071592	2486050	2486050	0.000
Xanthomonas oryzae pv. oryzae KACC10331	NC_006834	4941439	4749647	2390380	2390380	0.000
Xanthomonas oryzae pv. oryzae MAFF 311018	NC_007705	4940217	291921	2369950	2369950	0.000
Xanthomonas oryzae pv. oryzae PXO99A	NC_010717	5240075	5089053	2372945	2372945	0.000
Xylella fastidiosa 9a5c	NC_002488	2679306	142	1784870	1784870	0.000
Xylella fastidiosa M12	NC_010513	2475130	19	1181078	1176280	0.194
Xylella fastidiosa Temecula1	NC_004556	2519802	145	1134575	1134575	0.000
Yersinia pestis Angola	NC_010159	4504254	269	2382809	2382809	0.000
Yersinia pestis Antiqua	NC_008150	4702289	269	1824860	1824860	0.000
Yersinia pestis biovar Microtus str. 91001	NC_005810	4595065	20	2298550	2298550	0.000
Yersinia pestis CO92	NC_003143	4653728	270	2330540	2330540	0.000
Yersinia pestis KIM	NC_004088	4600755	20	2324409	2324409	0.000

Yersinia pestis Nepal516	NC_008149	4534590	269	1963950	1963950	0.000
Yersinia pestis Pestoides F	NC_009381	4517345	4456607	981535	981535	0.000
Yersinia pseudotuberculosis IP 31758	NC_009708	4723306	20	2153660	2153660	0.000
Yersinia pseudotuberculosis IP 32953	NC_006155	4744671	269	2592375	2592375	0.000
Yersinia pseudotuberculosis PB1/+	NC_010634	4695619	4660885	2511420	2511420	0.000

1. Origin and terminus as determined by pentamer analysis
