# Supplementary material for: Chromosome architecture constrains horizontal gene transfer in bacteria
Source: PLoS Genet. 2018 May 29;14(5):e1007421. doi: 10.1371/journal.pgen.1007421 (PMC5993296; doi:10.1371/journal.pgen.1007421)
Supplement: S6 Table — (PDF) [file pgen.1007421.s007.pdf]

Table S6. Correlation of gene data with distance from the replication terminus

		γ-Proteobacteria			α,β-Proteobacteria		Actinobacteria			Firmicutes			Bacteroidetes
		**Eco**	**Hin**	**Vch**	**Ccr**	**Bma**	**Msm**	**Blo**	**Cef**	**Bpu**	**Lre**	**Sag**	**Bvu**
Operon Length	m	2.918	-2.972	-3.731	-1.594	0.406	2.252	1.164	-0.688	2.514	-3.135	-2.786	5.804
	R^2	0.001	0.002	0.002	0.000	0.000	0.000	0.000	0.000	0.001	0.001	0.001	0.003
	P	0.077	0.177	0.091	0.297	0.807	0.148	0.570	0.719	0.198	0.234	0.257	0.004
Genes per Operon	m	0.001	-0.002	0.000	-0.001	0.002	0.001	-0.001	-0.001	0.002	-0.006	0.002	0.000
	R^2	0.000	0.001	0.000	0.000	0.001	0.000	0.000	0.000	0.000	0.004	0.001	0.000
	P	0.581	0.296	0.871	0.329	0.217	0.476	0.464	0.687	0.326	0.022	0.354	0.953
Measures of codon usage bias													
ACEu	m	0.002	0.002	0.001	0.000	0.001	0.001	0.000	0.000	0.000	0.001	0.001	0.000
	R^2	0.014	0.013	0.006	0.000	0.009	0.003	0.000	0.000	0.000	0.001	0.006	0.000
	P	5.09E-14	4.59E-06	2.55E-05	3.73E-01	3.63E-08	1.32E-05	5.43E-01	2.39E-01	6.25E-01	1.44E-01	3.63E-04	5.94E-01
Karlin's E	m	0.002	0.002	0.003	0.000	0.001	0.000	0.001	0.001	0.001	0.000	0.002	0.001
	R^2	0.006	0.005	0.011	0.000	0.006	0.001	0.000	0.001	0.004	0.000	0.008	0.003
	P	8.85E-07	4.46E-03	6.78E-08	2.35E-01	1.04E-05	5.33E-02	4.54E-01	8.45E-02	2.71E-04	5.14E-01	2.35E-05	1.28E-03
GCB	m	0.001	0.001	0.001	0.000	0.001	0.000	0.000	0.000	0.000	0.000	0.001	0.000
	R^2	0.012	0.012	0.006	0.000	0.010	0.003	0.000	0.000	0.000	0.002	0.006	0.000
	P	8.92E-13	9.76E-06	3.77E-05	3.53E-01	7.87E-09	2.47E-05	6.33E-01	3.60E-01	7.87E-01	9.05E-02	2.46E-04	9.67E-01
MELP	m	0.002	0.002	0.003	0.000	0.001	0.000	0.001	0.000	0.001	0.000	0.002	0.001
	R^2	0.012	0.007	0.010	0.000	0.011	0.002	0.002	0.000	0.003	0.000	0.008	0.003
	P	9.53E-13	5.20E-04	1.19E-07	5.51E-01	1.25E-09	1.28E-03	4.51E-02	2.90E-01	8.21E-04	4.63E-01	5.53E-05	1.07E-03

Distance between nearest inverted oligomers

Pentamers	m	1.306	-0.415	1.061	0.697	-0.285	-0.775	-0.313	-0.545	-0.749	-0.636	-0.202	2.191
	R^2	0.003	0.006	0.009	0.004	0.007	0.004	0.008	0.005	0.003	0.012	0.001	0.046
	P	0.426	0.517	0.313	0.388	0.467	0.300	0.433	0.406	0.504	0.311	0.768	0.002
Hexamers	m	0.182	-0.109	0.259	-0.163	-0.089	-0.069	-0.069	-0.065	-0.419	-0.220	-0.074	0.476
	R^2	0.002	0.010	0.015	0.009	0.037	0.002	0.007	0.004	0.022	0.034	0.005	0.039
	P	0.550	0.386	0.183	0.216	0.084	0.455	0.460	0.488	0.072	0.080	0.516	0.005
Heptamers	m	-0.047	-0.030	0.046	0.004	-0.015	-0.023	-0.016	-0.004	-0.111	0.026	0.019	0.107
	R^2	0.006	0.030	0.007	0.000	0.048	0.018	0.020	0.001	0.104	0.033	0.022	0.036
	P	0.352	0.226	0.365	0.825	0.091	0.097	0.272	0.792	0.001	0.200	0.359	0.007

m : slope of regression line between statistic and distance of gene/operon from the replication terminus. R^2 : square of pearson's correlation. P : *P*-value of the slope being different from zero (F-test); individual *P*-values are not corrected for multiple tests. Species : Eco, *Escherichia coli* MG1655; Hin, *Haemophilus influenzae* Rd KW20; Vch, *Vibrio cholerae* N16961; Cre, *Caulobacter crescentus* CB15; Bma, *Burkholderia mallei* NCTC 10229; Msm, *Mycobacterium smegmatis* MC2155; Blo, *Bifidobacterium longum* NCC2705; Fal, *Frankia alni* ACN14a; Cef, *Corynebacterium efficiens* YS-314; Bpu, *Bacillus pumilus* SAFR-032; Lre, *Lactobacillus reuteri* JCM 1112; Sag, *Streptococcus agalactiae* 2603V/R; Bvu, *Bacteroides vulgatus* ATCC 8482.
