# Supplementary material for: Chromosome architecture constrains horizontal gene transfer in bacteria
Source: PLoS Genet. 2018 May 29;14(5):e1007421. doi: 10.1371/journal.pgen.1007421 (PMC5993296; doi:10.1371/journal.pgen.1007421)
Supplement: S7 Table — (PDF) [file pgen.1007421.s008.pdf]

Table S7. Average bias of AIMS within donor fragments.

	Same Division		Different Division			Same Family		Different Family		
**Division**	**N**	**Bias[1]**	**N**	**Bias**	**Delta**	**N**	**Bias**	**N**	**Bias**	**Delta**
Actinobacteria	486	57.36	4527	56.94	0.42	42	59.33	444	57.17	2.16
Alphaproteobacteria	660	56.74	4910	56.87	-0.13	51	58.10	609	56.63	1.47
Bacteroidetes	54	56.31	1617	56.09	0.22	8	62.45	46	55.25	7.21
Betaproteobacteria	185	57.88	2600	57.67	0.21	26	59.70	159	57.58	2.13
Chlamydiae	12	60.76	1102	55.61	5.15	10	62.04	2	54.33	7.71
Cyanobacteria	69	54.34	1602	55.19	-0.85	4	57.71	65	54.13	3.58
Deltaproteobacteria	81	56.86	1590	56.27	0.59	17	61.10	64	55.73	5.37
Epsilonproteobacteria	30	57.78	1084	56.93	0.86	12	59.19	18	56.84	2.35
Firmicutes	1344	65.50	7568	56.12	9.38	167	66.65	1177	65.34	1.31
Gammaproteobacteria	2369	58.54	10442	57.46	1.07	284	59.98	2085	58.34	1.65
Spirochaetes	24	64.80	1647	57.85	6.95	12	68.40	12	61.21	7.19
Tenericutes	51	58.21	1620	54.76	3.45	28	59.20	23	56.99	2.21

1. Bias is average strand-bias of recipient AIMS in donor genomes; genomes are partitioned according to the phylogenetic relationship between donors and recipients. Organisms in the same family as a recipient are always better donors, regardless of which division we are looking at. In all but two cases, organisms in the same division are better donors than organisms from other divisions. The values of N are the number of comparisons.
