# Supplementary figures and images for: Chromosome architecture constrains horizontal gene transfer in bacteria
Source: PLoS Genet. 2018 May 29;14(5):e1007421. doi: 10.1371/journal.pgen.1007421 (PMC5993296; doi:10.1371/journal.pgen.1007421)

Figure S1

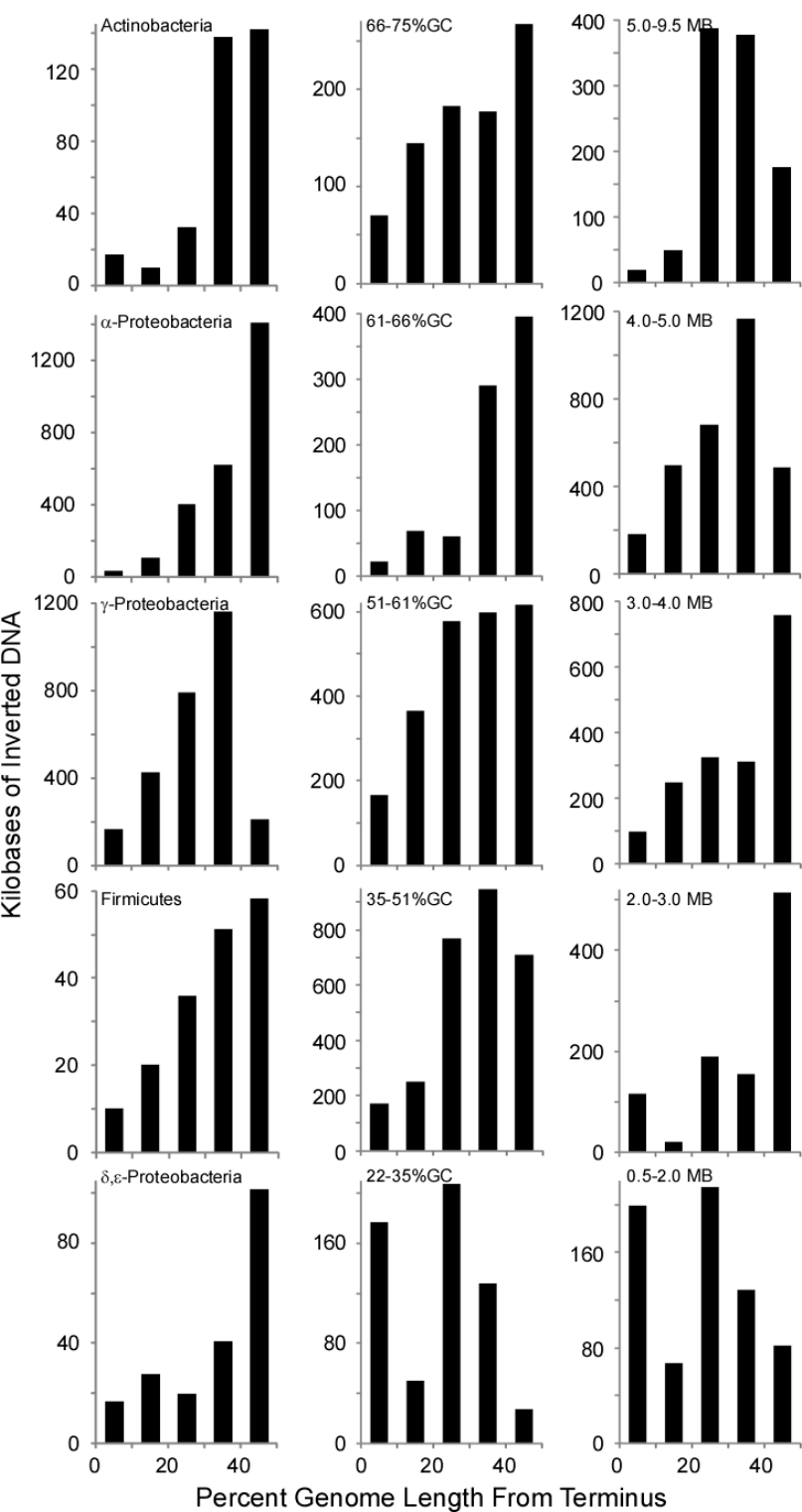

Supplement: S1 Fig — A total of 634 inversions were identified in 159 pairwise comparisons of 214 separate completely sequenced genomes (See S2 Table for details). All data are plotted as % genome distance of the midpoint of the inversion from the replication terminus. The total length of DNA inverted plotted by genome position across all genomes included in the analysis. (PDF) [file pgen.1007421.s009.pdf]

Figure S2

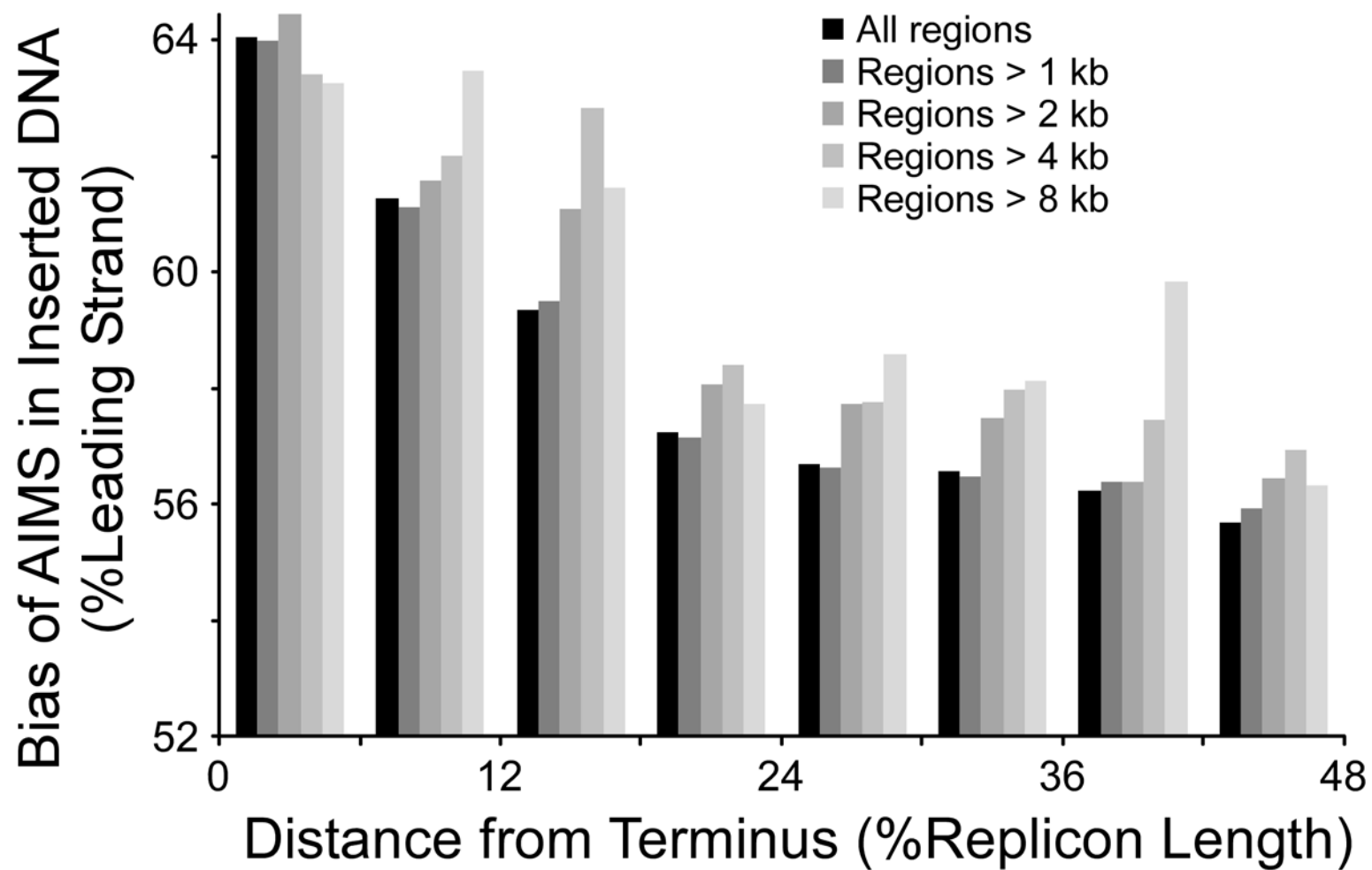

Supplement: S2 Fig — Strand-bias is assessed for insertions with within chromosomal regions with increasing distance from the replication terminus. Black bars depict average strand bias for all genes (data also presented in Fig 5). Gray bars depict average strand bias for subsets of data whereby the clusters of contiguous inserted genes analysed must lie in regions larger than 1kb, 2 kb, 4 kb or 8 kb. (PDF) [file pgen.1007421.s010.pdf]

Figure S3

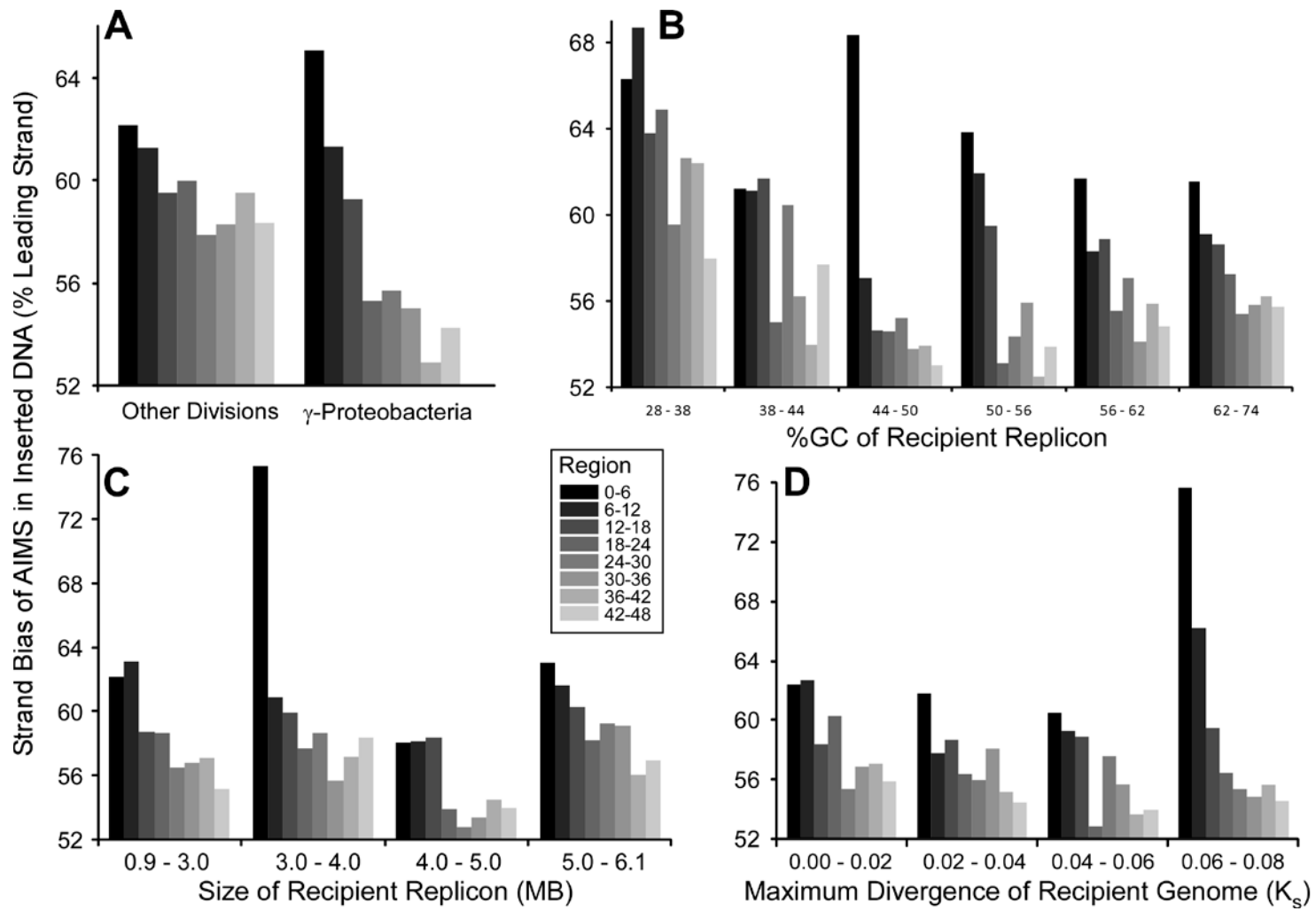

Supplement: S3 Fig — Strand-bias is assessed for insertions with within chromosomal regions with increasing distance from the replication terminus. A. Organisms are segregated into γ-Proteobacteria and other divisions; other divisions lack the sample size to assay individually. B. Organisms are segregated by GC content. C. Organisms are segregated by genome size. D. Organisms are segregated by the average divergence at synonymous sites between the organisms bearing the insertion and the most closely-related genome which lacks the insertion, thus placing an upper bound on the age of the insertion within the recipient genome. (PDF) [file pgen.1007421.s011.pdf]
